# Supplementary figures and images for: A meta-evaluation of the quality of reporting and execution in ecological meta-analyses
Source: PLoS One. 2023 Oct 12;18(10):e0292606. doi: 10.1371/journal.pone.0292606 (PMC10569516; doi:10.1371/journal.pone.0292606)

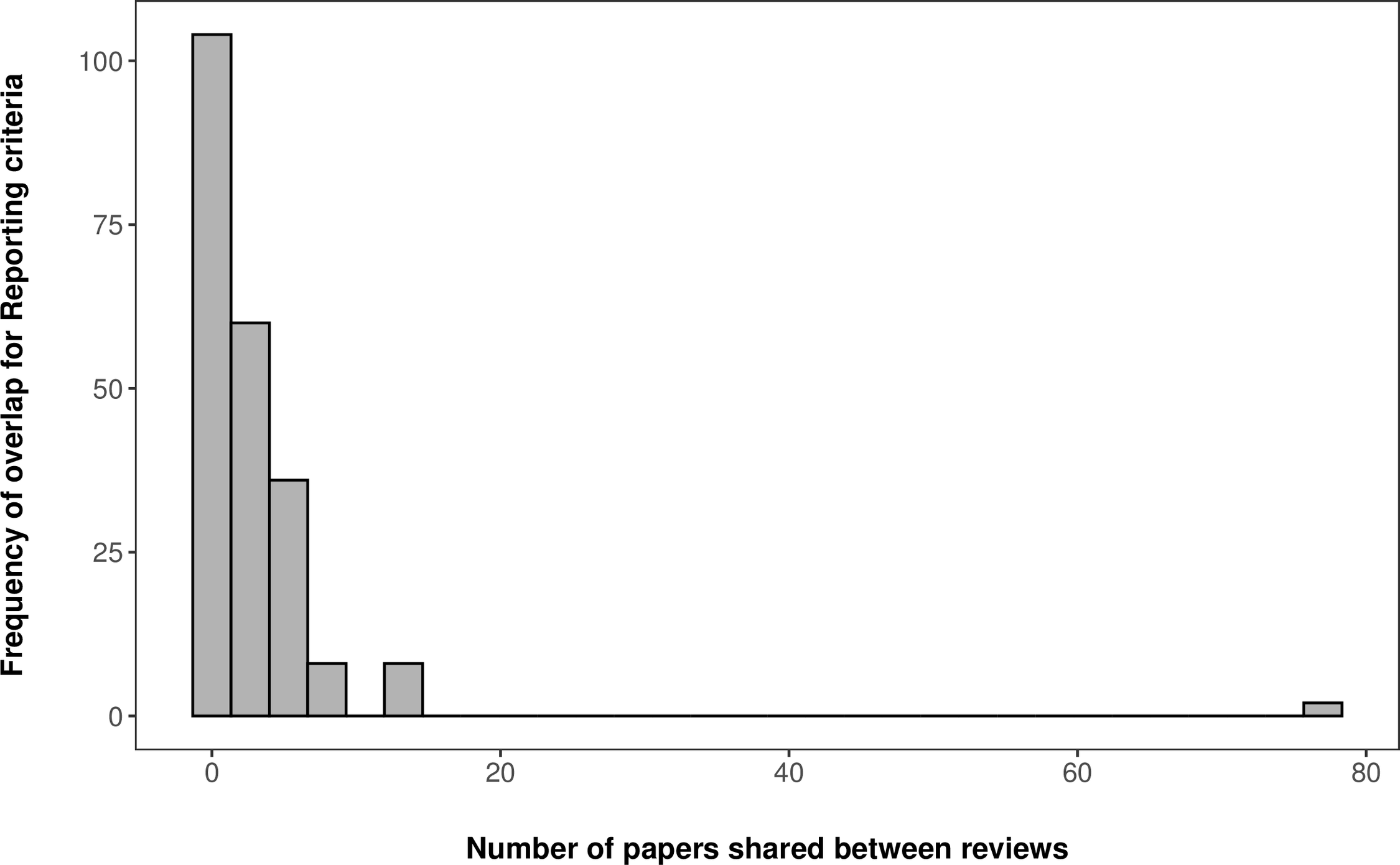

Supplement: S1 Fig — Distribution of the number of papers shared between reviews for all the Reporting criteria combined. (TIF) [file pone.0292606.s002.tif]

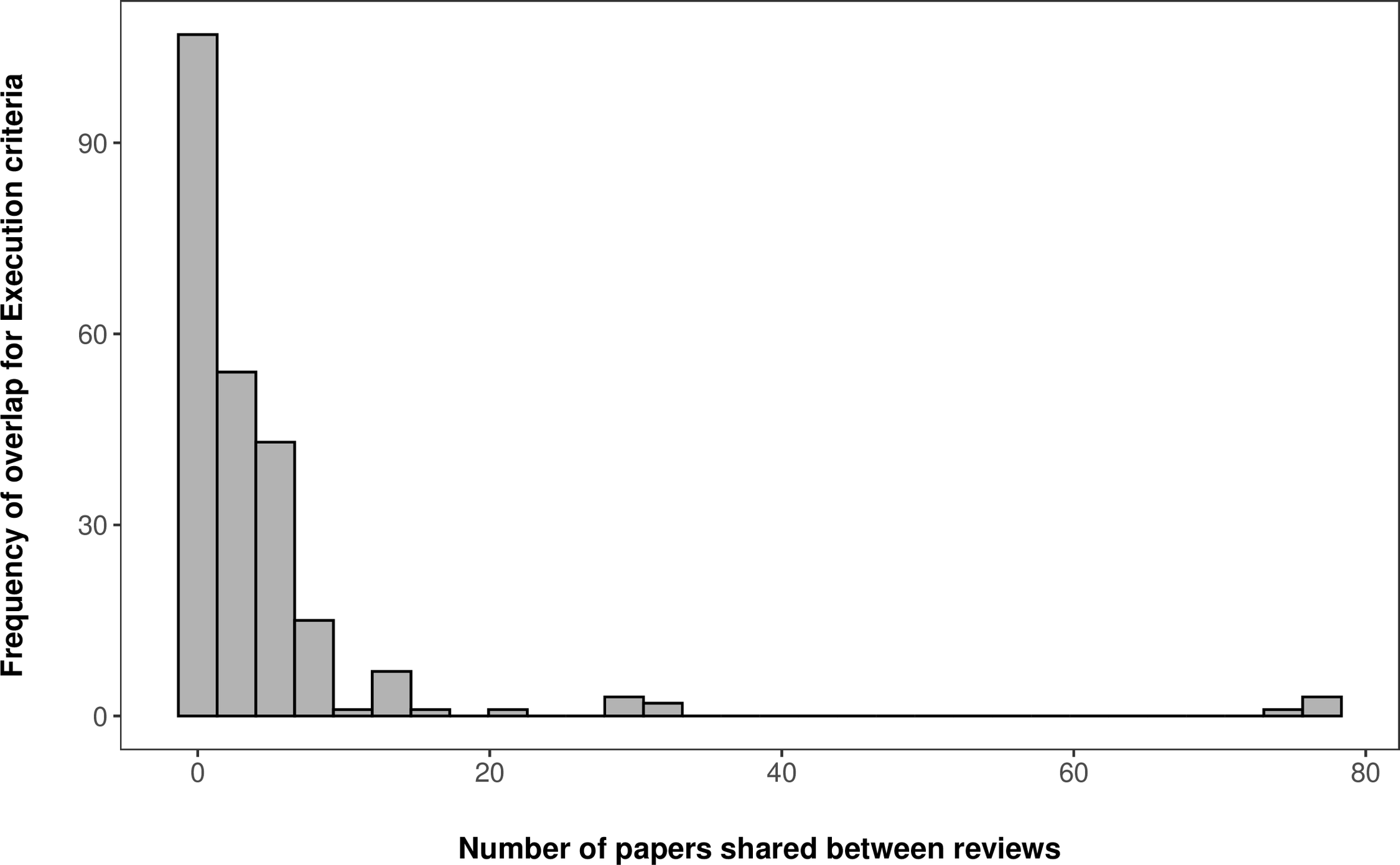

Supplement: S2 Fig — Distribution of the number of papers shared between reviews for all the Execution criteria combined. (TIF) [file pone.0292606.s003.tif]

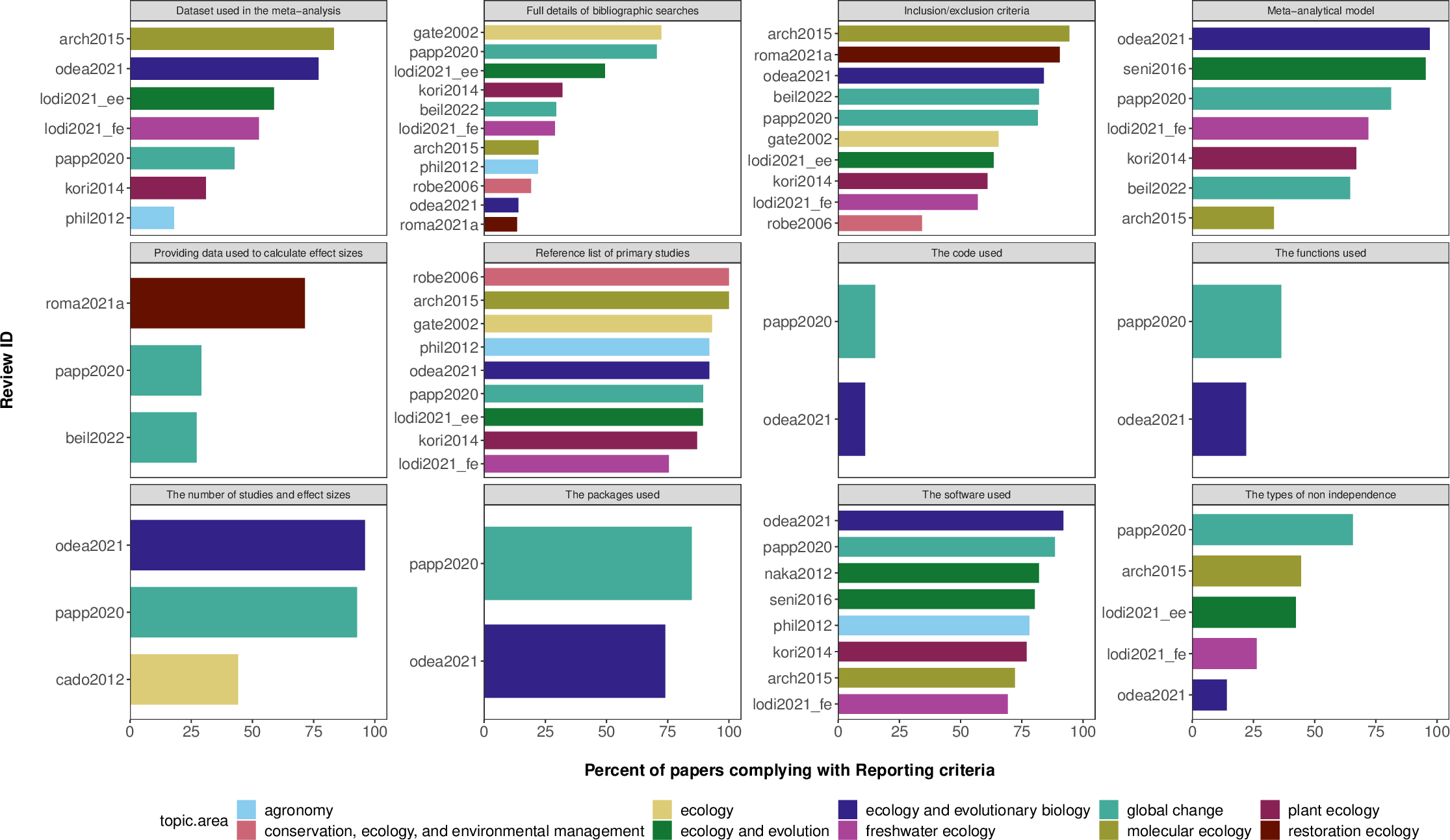

Supplement: S3 Fig — The percent of papers complying with each Reporting criterion is plotted for each review paper. The colors indicate different subdisciplines of the review papers. The Review ID corresponds to the papers listed in Table 1 of the main manuscript. (TIF) [file pone.0292606.s004.tif]

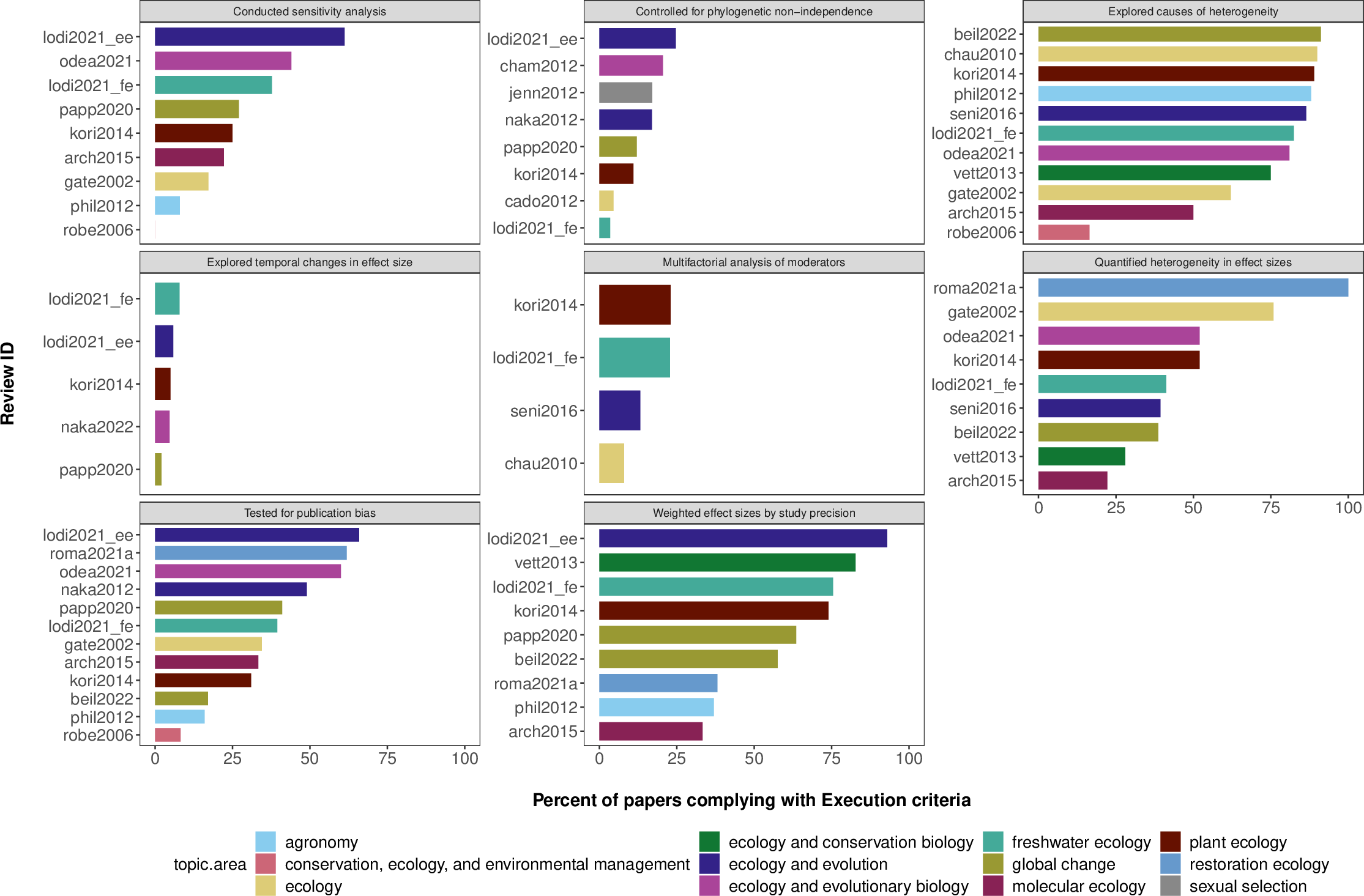

Supplement: S4 Fig — The percent of papers complying with each Execution criterion is plotted for each review paper. The colors indicate different subdisciplines of the review papers. The Review ID corresponds to the papers listed in Table 1 of the main manuscript. (TIF) [file pone.0292606.s005.tif]

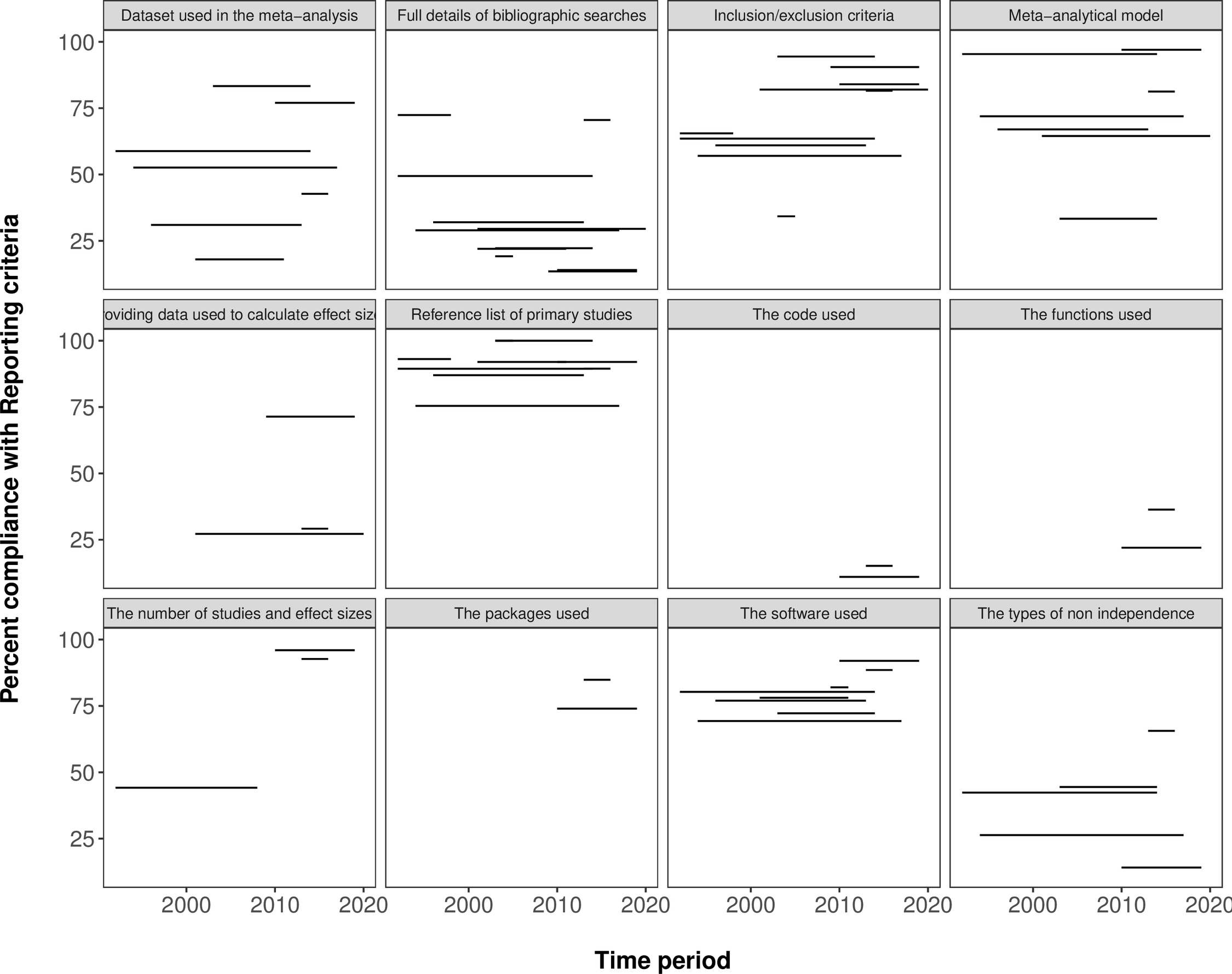

Supplement: S5 Fig — Each panel represents a Reporting criterion. The line segment indicates the time period covered by each of the review papers that addressed a particular criterion. (TIF) [file pone.0292606.s006.tif]

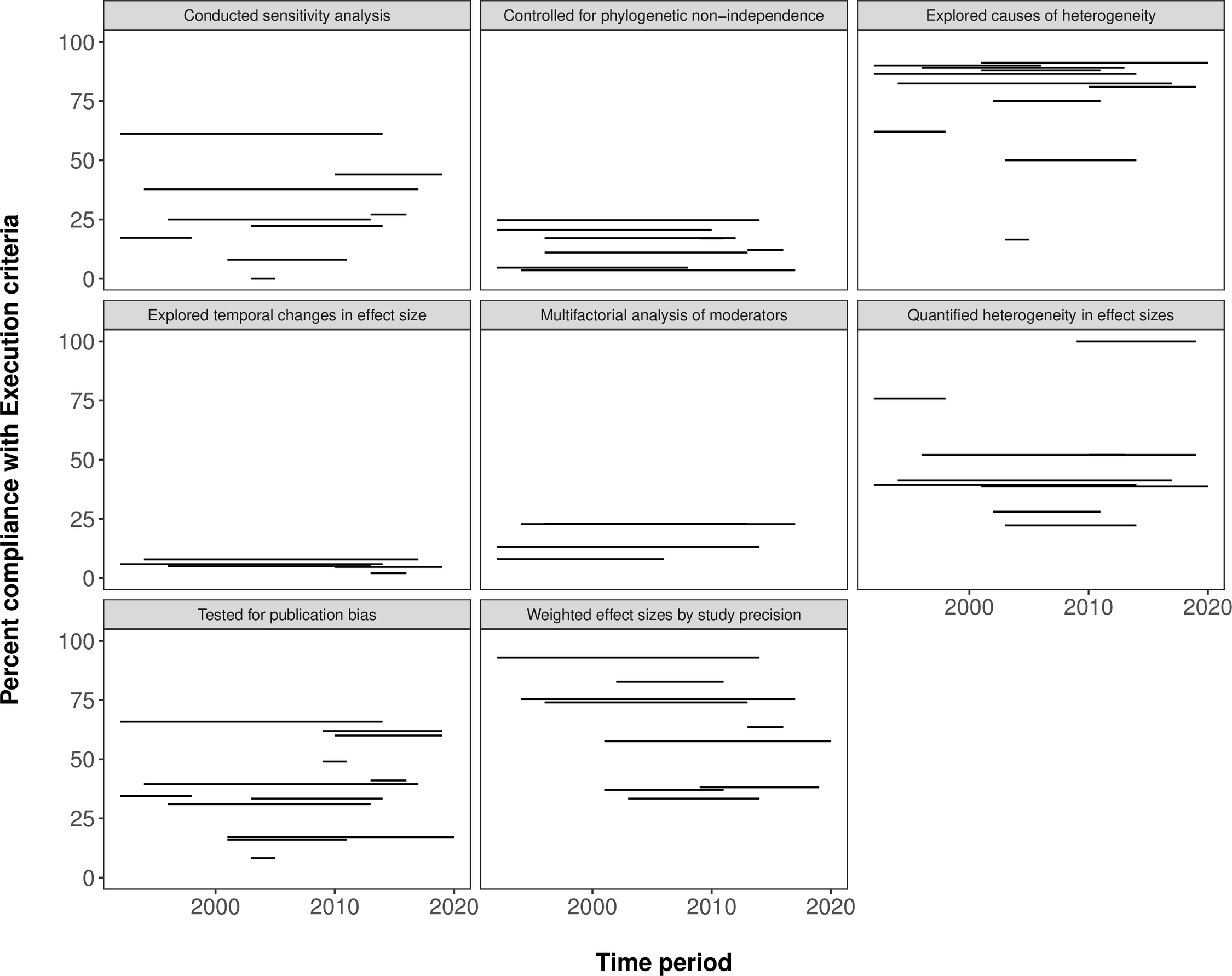

Supplement: S6 Fig — Each panel represents an Execution criterion. The line segment indicates the time period covered by each of the review papers that addressed a particular criterion. (TIF) [file pone.0292606.s007.tif]
