# Supplementary material for: A meta-evaluation of the quality of reporting and execution in ecological meta-analyses
Source: PLoS One. 2023 Oct 12;18(10):e0292606. doi: 10.1371/journal.pone.0292606 (PMC10569516; doi:10.1371/journal.pone.0292606)
Supplement: S1 Appendix — Additional details and R code for paper screening using the package metagear. (PDF) [file pone.0292606.s008.pdf]

Appendix S1 - Paper screening

Paula Pappalardo, Chao Song, Bruce A. Hungate, Craig W. Osenberg

From: A meta-evaluation of the quality of reporting and execution in ecological meta-analyses

Additional details and code for paper screening

We screened the 751 abstracts of citation downloaded from Web Of Science using the package **metagear** [13]. We used the function *effort_initialize()* to add columns "study_id", "reviewers" and "include". After that, the *abstract_screener()* function brings up an interactive window to rate the abstract for inclusion or not, the options are: "yes", "maybe", and "no". The rating column gets added. The package adds a new column in the citation data with this decision. It can also split the effort among different coauthors; if so, it is necessary to include a column with the reviewer/s name and assign reviewer's effort (see code below). Paper screening and data extraction from the reviews of meta-analysis was done by Paula Pappalardo; additional data extraction for the non-independence section was done by Chao Song.

When in doubt if to include a paper, they were tagged as "maybe" and the full text was evaluated. Here are the R code and **metagear** specific functions we used:

```
# load data downloaded from Web of Science and select columns we need

wos <- as.data.frame(read_excel("WOS/meta-analysis_WOS_751.xls",
                                 sheet= "savedrecs", range= cell_cols("A:BT"))) %>%
  select("Publication Type", "Authors", "Article Title",
                     "Source Title", "Volume", "Issue", "Start Page",
                     "End Page", "DOI", "Publication Date", "Publication Year",
                     "Abstract", "WoS Categories")

# rename columns

names(wos) <- c("pub_type", "authors", "title", "pub_name","vol", "issue",
                "start_page", "end_page", "doi", "pub_date", "pub_year",
                "abstract","wos_categ")
```

```
library(metagear)

# prepare the file for the screening effort

wos_scan <- effort_initialize(wos)

# save file with the IDs as a backup

write.csv(wos_scan, "WOS/wosWithIds.csv")

# randomly distribute screening effort to a team
```

```r
theTeam <- c("Paula")
theRefs_unscreened <- effort_distribute(wos_scan, reviewers = theTeam,
    effort = c(100), save_split = T)

# start the abstract viewer to do first pass

abstract_screener("effort_Paula.csv", aReviewer = "Paula",
    abstractColumnName = "abstract", titleColumnName = "title")

# get the summary of your work

theRefs_screened <- effort_merge()
sum.scan <- effort_summary(theRefs_screened)

# the effort file got saved by default in the main project, I moved it to the WOS folder
```

```r
# load revision file

refs <- read.csv("WOS/effort_Paula.csv")

# subset by inclusion decision

theRefs_included <- refs[which(refs$INCLUDE == "YES" | refs$INCLUDE ==
    "MAYBE"), ]

# try getting pdfs from R

PDFs_collect(theRefs_included, DOIcolumn = "doi", FileNamecolumn = "STUDY_ID",
    directory = "C:/Users/Paula/Dropbox/Meta-analysis/ReportingQuality/WOS/pdfs")
```

We manually added the papers found with other sources to the "effort_Paula.csv", and provide the file *"papers-screened_Final-classification.csv"* as supplementary data. This file has the final list of papers screened, the final decision, and the reasons to exclude papers after reading the full text.
