# Supplementary material for: A meta-evaluation of the quality of reporting and execution in ecological meta-analyses
Source: PLoS One. 2023 Oct 12;18(10):e0292606. doi: 10.1371/journal.pone.0292606 (PMC10569516; doi:10.1371/journal.pone.0292606)
Supplement: S2 Appendix — Additional details and R code used to calculate overlap between review papers, including overlap matrices for each criterion and R code for S1 and S2 Figs. (PDF) [file pone.0292606.s009.pdf]

Appendix S2

Paula Pappalardo, Chao Song, Bruce A. Hungate, Craig W. Osenberg

From: A meta-evaluation of the quality of reporting and execution in ecological meta-analyses

Setup

```
knitr::opts_chunk$set(echo = T, eval = T, warning = F,
                      message = F, comment = "")

# load libraries we need

library(kableExtra)
library(tidyverse)
library(ggcharts)
library(rcartocolor)
library(readxl)
library(flextable)
```

```
# load objects we need, generated with the R code provided

load("objects/reporting.R")
load("objects/execution.R")
load("objects/toplot.R")

# nice format for plots

niceplot <- theme_bw() +
  theme(panel.grid.major = element_blank(),
        panel.grid.minor = element_blank(),
        axis.title.x = element_text(face = "bold", size = 12,
                                    margin = margin(t = 20, r = 0, b = 0, l = 0)),
        axis.title.y = element_text(face = "bold", size = 12,
                                    margin = margin(t = 0, r = 20, b = 0, l = 0)),
        axis.text.x = element_text(size = 11),
        axis.text.y = element_text(size = 11),
        legend.text = element_text(size = 11),
        legend.title = element_text(size = 11),
        legend.position = "right")
```

Overlap between review papers

We compiled the list of references analyzed by each review paper, by extracting them from the main text, the references section, supplementary materials, or by directly emailing the authors. For the following papers we were unable to obtain the list of references, and were not included in the tables of overlap:

- The references for Chaudhary et al. [21] paper were not accessible, we checked their supplementary information and data packages, but the web link gives an error. We also contacted the authors and had no answer.
- The references for Nakagawa & Santos [23] were not provided. We emailed the authors, S. Nakagawa promptly responded, and contacted E. Santos. The authors apologized for being unable to find the data after so many years.

In some cases, the journal names were not identical between lists (e.g., American Naturalist versus The American Naturalist), or had different spellings (e.g., 'Soil Biology & Biochemistry' versus 'Soil, Biology & Biochemistry'). Before analysis, we standardized all the journals names to the long format, and cross checked (and fixed when necessary) the journal names across reviews. We also checked for authors names that had different spelling across reviews (e.g., Castro-vila, vs Castro Vila) and followed the spelling used by most review authors. After the quality check, we created a **review code** by combining: 1) the first author last name (in caps), 2) the year of publication, and 3) the journal (in caps). We standardized the text to uppercase to correct for inconsistencies in spelling (e.g., Van Groenigen versus van Groenigen for authors, or PLoS ONE versus PLOS ONE for journals). Here an example of a final reference id: BYERS_1999_ANNUAL REVIEW OF ECOLOGY AND SYSTEMATICS. The list of references is available in the supplementary data file "DataFiles_list-of-references-in-Reviews".

```r
# function to select columns of interest

selectColumns <- function(mydf){
  mydf_ed <- mydf %>% select(first_author, year, journal)
  return(mydf_ed)
}

# function to add a standarized reference ID to compare accross reviews
addID <- function(mydf){
  mydf_ed <- mydf %>%
    mutate(ref_id = paste(str_to_upper(first_author), year,
                          str_to_upper(journal), sep = "_"),
           journal = str_to_upper(journal))
  return(mydf_ed)
}


# load short journals dictionary

journal_dic <- read.csv("data/DataFiles_journal-names-dictionary.csv", as.is = T)
```

```r
# load references for each publication

arch <- as.data.frame(read_excel("data/DataFiles_list-of-references-in-Reviews.xlsx",
                                 sheet= "arch2015", range= cell_cols("A:D"))) %>%
  selectColumns() %>%
```

```
  addID()

cado <- as.data.frame(read_excel("data/DataFiles_list-of-references-in-Reviews.xlsx",
                                 sheet= "cado2012", range= cell_cols("A:D"))) %>%
  selectColumns() %>%
  addID()

cham <- as.data.frame(read_excel("data/DataFiles_list-of-references-in-Reviews.xlsx",
                                 sheet= "cham2012", range= cell_cols("A:C"))) %>%
  addID()

gate <- as.data.frame(read_excel("data/DataFiles_list-of-references-in-Reviews.xlsx",
                                 sheet= "gate2002", range= cell_cols("A:C"))) %>%
  addID()

jenn <- as.data.frame(read_excel("data/DataFiles_list-of-references-in-Reviews.xlsx",
                                 sheet= "jenn2012", range= cell_cols("A:B"))) %>%
  left_join(journal_dic, by = "journal_short") %>%
  rowwise() %>%
  mutate(year = as.numeric(str_extract(reference, "\\d{4}")[[1]]),
         first_author = str_split_fixed(reference, " ", 2)[,1]) %>%
  ungroup() %>%
  selectColumns() %>% addID()

kori <- as.data.frame(read_excel("data/DataFiles_list-of-references-in-Reviews.xlsx",
                                 sheet= "kori2012", range= cell_cols("A:C"))) %>%
  addID()

odea <- as.data.frame(read_excel("data/DataFiles_list-of-references-in-Reviews.xlsx",
                                 sheet= "odea2021", range= cell_cols("A:C"))) %>%
  addID()

papp <- as.data.frame(read_excel("data/DataFiles_list-of-references-in-Reviews.xlsx",
                                 sheet= "papp2020", range= cell_cols("A:C"))) %>%
  addID()

phil<- as.data.frame(read_excel("data/DataFiles_list-of-references-in-Reviews.xlsx",
                                sheet= "phil2012", range= cell_cols("A:C"))) %>%
  left_join(journal_dic, by = "journal_short") %>%
  selectColumns() %>% addID()

robe <- as.data.frame(read_excel("data/DataFiles_list-of-references-in-Reviews.xlsx",
                                 sheet= "robe2006", range= cell_cols("A:C"))) %>%
  addID()

seni <- as.data.frame(read_excel("data/DataFiles_list-of-references-in-Reviews.xlsx",
                                 sheet= "seni2016", range= cell_cols("A:C"))) %>%
  addID()

vett <- as.data.frame(read_excel("data/DataFiles_list-of-references-in-Reviews.xlsx",
                                 sheet= "vett2013", range= cell_cols("A:C"))) %>%
  addID()
```

```
lodi_fe <- as.data.frame(read_excel("data/DataFiles_list-of-references-in-Reviews.xlsx",
                                    sheet= "lodi2021_fe", range= cell_cols("A:C"))) %>%
  mutate(first_author =  str_split_fixed(Citation, ",", 2)[,1],
         journal = ifelse(journal == "The American Naturalist",
                          "American Naturalist", journal)) %>%
  addID()

lodi_ee <- as.data.frame(read_excel("data/DataFiles_list-of-references-in-Reviews.xlsx",
                                    sheet= "lodi2021_ee", range= cell_cols("A:C"))) %>%
  addID()

beil <- as.data.frame(read_excel("data/DataFiles_list-of-references-in-Reviews.xlsx",
                                 sheet= "beil2022", range= cell_cols("A:D"))) %>%
  addID()

roma_a <- as.data.frame(read_excel("data/DataFiles_list-of-references-in-Reviews.xlsx",
                  sheet= "roma2021a", range= cell_cols("A:D"))) %>%
  addID()

roma_b <- as.data.frame(read_excel("data/DataFiles_list-of-references-in-Reviews.xlsx",
                  sheet= "roma2021b", range= cell_cols("A:C"))) %>%
  rowwise() %>%
  mutate(year = as.numeric(str_extract(Citation, "\\d{4}")[[1]])) %>%
  ungroup() %>%
  addID()
```

Compare references between the two Romanelli papers:

```
# number of papers shared
length(union(roma_a$ref_id, roma_b$ref_id))

# number of unique papers in Romanelli_a
length(setdiff(roma_a$ref_id, roma_b$ref_id))

# number of unique papers in Romanelli_b
length(setdiff(roma_b$ref_id, roma_a$ref_id))
```

```
# Compile all first author names

allnames <- c(arch$first_author, cado$first_author, cham$first_author, gate$first_author,
              jenn$first_author, kori$first_author, odea$first_author, papp$first_author,
              phil$first_author, robe$first_author, seni$first_author, vett$first_author,
              lodi_fe$first_author, beil$first_author, roma_a$first_author)

View(data.frame(sort(unique(allnames))))

alljournals <- c(arch$journal, cado$journal, cham$journal, gate$journal,
              jenn$journal, kori$journal, odea$journal, papp$journal,
              phil$journal, robe$journal, seni$journal, vett$journal,
              lodi_fe$journal, beil$journal, roma_a$journal)

View(data.frame(sort(unique(alljournals))))
```

```r
load("objects/toplot.R")

# prepare list of criteria

rep_criteria <- toplot %>%
  filter(criteria != "Type of uncertainty interval was described") %>%
  filter(criteria != "Describe effect size used") %>%
  filter(criteria != "The number of studies excluded at each stage of screening") %>%
  filter(criteria.type == "Reporting") %>%
  distinct(criteria)
repvec <- rep_criteria$criteria

exe_criteria <- toplot %>%
  filter(criteria.type == "Execution") %>%
  filter(!criteria == "Standard metrics") %>%
  distinct(criteria)
exevec <- exe_criteria$criteria

# prepare list of references for each review paper

vecs <- list(arch$ref_id, cado$ref_id, cham$ref_id, gate$ref_id,
          jenn$ref_id, kori$ref_id, odea$ref_id, papp$ref_id,
          phil$ref_id, robe$ref_id, seni$ref_id, vett$ref_id,
          lodi_fe$ref_id, lodi_ee$ref_id, beil$ref_id, roma_a$ref_id)

names(vecs) <- c("arch2015", "cado2012", "cham2012", "gate2002", "jenn2012",
                 "kori2014", "odea2021", "papp2020", "phil2012", "robe2006",
                 "seni2016", "vett2013", "lodi2021_fe", "lodi2021_ee",
                 "beil2022", "roma2021a")

# function to get matrix of overlap

getPercentOverlapMatrix <- function(toplot, criterion, vecs){
   # separate the criterion of interest
    thiscriteria <- toplot %>%
    filter(criteria == criterion)
    # subset the vecs list to papers that measure this criterion
    shortlist <- vecs[names(vecs) %in% unique(thiscriteria$paper.id)]
    # create matrix
    mymat <- matrix(ncol = length(shortlist),
                    nrow = length(shortlist),
                    dimnames = list(names(shortlist), names(shortlist)))
  # run loop to calculate the differences between papers
  for(i in 1:length(shortlist)){
    thisvec <- shortlist[[i]]
    for(j in 1:length(shortlist)){
      mymat[i, j] <- length(intersect(thisvec, shortlist[[j]]))
      }
  }
  return(mymat)
}
```

Because the overlap only matters within each criteria used to compare review papers, we calculated the

matrix of paper overlap for each criteria.

Matrix of overlap for Reporting criteria

Full details of bibliographic searches:

```r
# calculate number of papers shared by reviews
rp1 <- getPercentOverlapMatrix(toplot, criterion = repvec[1], vecs = vecs)
rp1
```

```
            arch2015 gate2002 kori2014 odea2021 papp2020 phil2012 robe2006
arch2015          18        0        2        0        0        0        0
gate2002           0       29        2        0        0        0        0
kori2014           2        2      322        5        3        5        2
odea2021           0        0        5      101        3        0        0
papp2020           0        0        3        3       96        0        0
phil2012           0        0        5        0        0       55        0
robe2006           0        0        2        0        0        0       73
lodi2021_fe        0        4        5        2        3        0        0
lodi2021_ee        2        4        5        4        3        0        1
beil2022           0        0        6        1       13        6        0
roma2021a          0        0        9        0        2        0        0
            lodi2021_fe lodi2021_ee beil2022 roma2021a
arch2015              0           2        0         0
gate2002              4           4        0         0
kori2014              5           5        6         9
odea2021              2           4        1         0
papp2020              3           3       13         2
phil2012              0           0        6         0
robe2006              0           1        0         0
lodi2021_fe         114           1        0         2
lodi2021_ee           1          86        1         0
beil2022              0           1      217         7
roma2021a             2           0        7        65
```

```r
# extract overlap elements
rp1_o <- rp1[lower.tri(rp1)]
```

Inclusion/exclusion criteria:

```r
# calculate number of papers shared by reviews
rp2 <- getPercentOverlapMatrix(toplot, criterion = repvec[2], vecs = vecs)
rp2
```

```
            arch2015 gate2002 kori2014 odea2021 papp2020 robe2006 lodi2021_fe
arch2015          18        0        2        0        0        0           0
gate2002           0       29        2        0        0        0           4
kori2014           2        2      322        5        3        2           5
odea2021           0        0        5      101        3        0           2
papp2020           0        0        3        3       96        0           3
robe2006           0        0        2        0        0       73           0
lodi2021_fe        0        4        5        2        3        0         114
```

```
lodi2021_ee        2        4        5        4        3        1          1
beil2022           0        0        6        1       13        0          0
roma2021a          0        0        9        0        2        0          2
            lodi2021_ee beil2022 roma2021a
arch2015              2        0         0
gate2002              4        0         0
kori2014              5        6         9
odea2021              4        1         0
papp2020              3       13         2
robe2006              1        0         0
lodi2021_fe           1        0         2
lodi2021_ee          86        1         0
beil2022              1      217         7
roma2021a             0        7        65
```

```
# extract overlap elements
rp2_o <- rp2[lower.tri(rp2)]
```

Meta-analytical model:

```
# calculate number of papers shared by reviews
rp3 <- getPercentOverlapMatrix(toplot, criterion = repvec[3], vecs = vecs)
rp3
```

```
            arch2015 kori2014 odea2021 papp2020 seni2016 lodi2021_fe beil2022
arch2015          18        2        0        0        3           0        0
kori2014           2      322        5        3       77           5        6
odea2021           0        5      101        3       12           2        1
papp2020           0        3        3       96       13           3       13
seni2016           3       77       12       13      325           8        7
lodi2021_fe        0        5        2        3        8         114        0
beil2022           0        6        1       13        7           0      217
```

```
# extract overlap elements
rp3_o <- rp3[lower.tri(rp3)]
```

The software used:

```
# calculate number of papers shared by reviews
rp4 <- getPercentOverlapMatrix(toplot, criterion = repvec[4], vecs = vecs)
rp4
```

```
            arch2015 kori2014 odea2021 papp2020 phil2012 seni2016 lodi2021_fe
arch2015          18        2        0        0        0        3           0
kori2014           2      322        5        3        5       77           5
odea2021           0        5      101        3        0       12           2
papp2020           0        3        3       96        0       13           3
phil2012           0        5        0        0       55        6           0
seni2016           3       77       12       13        6      325           8
lodi2021_fe        0        5        2        3        0        8         114
```

```r
# extract overlap elements
rp4_o <- rp4[lower.tri(rp4)]
```

Reference list of primary studies:

```r
# calculate number of papers shared by reviews
rp5 <- getPercentOverlapMatrix(toplot, criterion = repvec[5], vecs = vecs)
rp5
```

```
             arch2015 gate2002 kori2014 odea2021 papp2020 phil2012 robe2006
arch2015           18        0        2        0        0        0        0
gate2002            0       29        2        0        0        0        0
kori2014            2        2      322        5        3        5        2
odea2021            0        0        5      101        3        0        0
papp2020            0        0        3        3       96        0        0
phil2012            0        0        5        0        0       55        0
robe2006            0        0        2        0        0        0       73
lodi2021_fe         0        4        5        2        3        0        0
lodi2021_ee         2        4        5        4        3        0        1
             lodi2021_fe lodi2021_ee
arch2015               0           2
gate2002               4           4
kori2014               5           5
odea2021               2           4
papp2020               3           3
phil2012               0           0
robe2006               0           1
lodi2021_fe          114           1
lodi2021_ee            1          86
```

```r
# extract overlap elements
rp5_o <- rp5[lower.tri(rp5)]
```

Dataset used in the meta-analysis:

```r
# calculate number of papers shared by reviews
rp6 <- getPercentOverlapMatrix(toplot, criterion = repvec[6], vecs = vecs)
rp6
```

```
             arch2015 kori2014 odea2021 papp2020 phil2012 lodi2021_fe
arch2015           18        2        0        0        0           0
kori2014            2      322        5        3        5           5
odea2021            0        5      101        3        0           2
papp2020            0        3        3       96        0           3
phil2012            0        5        0        0       55           0
lodi2021_fe         0        5        2        3        0         114
lodi2021_ee         2        5        4        3        0           1
             lodi2021_ee
arch2015               2
kori2014               5
odea2021               4
papp2020               3
```

```
phil2012              0
lodi2021_fe           1
lodi2021_ee          86
```

```r
# extract overlap elements
rp6_o <- rp6[lower.tri(rp6)]
```

The types of non independence:

```r
# calculate number of papers shared by reviews
rp7 <- getPercentOverlapMatrix(toplot, criterion = repvec[7], vecs = vecs)
rp7
```

```
            arch2015 odea2021 papp2020 lodi2021_fe lodi2021_ee
arch2015          18        0        0           0           2
odea2021           0      101        3           2           4
papp2020           0        3       96           3           3
lodi2021_fe        0        2        3         114           1
lodi2021_ee        2        4        3           1          86
```

```r
# extract overlap elements
rp7_o <- rp7[lower.tri(rp7)]
```

The number of studies and effect sizes:

```r
# calculate number of papers shared by reviews
rp8 <- getPercentOverlapMatrix(toplot, criterion = repvec[8], vecs = vecs)
rp8
```

```
         cado2012 odea2021 papp2020
cado2012      240        0        0
odea2021        0      101        3
papp2020        0        3       96
```

```r
# extract overlap elements
rp8_o <- rp8[lower.tri(rp8)]
```

The packages used:

```r
# calculate number of papers shared by reviews
rp9 <- getPercentOverlapMatrix(toplot, criterion = repvec[9], vecs = vecs)
rp9
```

```
         odea2021 papp2020
odea2021      101        3
papp2020        3       96
```

```r
# extract overlap elements
rp9_o <- rp9[lower.tri(rp9)]
```

The functions used:

```
# calculate number of papers shared by reviews
rp10 <- getPercentOverlapMatrix(toplot, criterion = repvec[10], vecs = vecs)
rp10
```

```
         odea2021 papp2020
odea2021      101        3
papp2020        3       96
```

```
# extract overlap elements
rp10_o <- rp10[lower.tri(rp10)]
```

The code used:

```
# calculate number of papers shared by reviews
rp11 <- getPercentOverlapMatrix(toplot, criterion = repvec[11], vecs = vecs)
rp11
```

```
         odea2021 papp2020
odea2021      101        3
papp2020        3       96
```

```
# extract overlap elements
rp11_o <- rp11[lower.tri(rp11)]
```

Providing data used to calculate effect sizes:

```
# calculate number of papers shared by reviews
rp12 <- getPercentOverlapMatrix(toplot, criterion = repvec[12], vecs = vecs)
rp12
```

```
          papp2020 beil2022 roma2021a
papp2020        96       13         2
beil2022        13      217         7
roma2021a        2        7        65
```

```
# extract overlap elements
rp12_o <- rp12[lower.tri(rp12)]
```

This is the summary of the vector including all the number of papers shared for all criteria:

```
allrep <-c(rp1_o, rp2_o, rp3_o, rp4_o, rp5_o, rp6_o, rp7_o, rp8_o,
           rp9_o, rp10_o, rp11_o, rp12_o)

summary(allrep)
```

```
   Min. 1st Qu.  Median    Mean 3rd Qu.    Max. 
  0.000   0.000   2.000   3.009   3.000  77.000 
```

This is the distribution of overlap for all Reporting criteria combined:

```r
# create dataframe to plot with ggplot

allrep_df <- data.frame(overlap = allrep)

# make histogram

hist_rep <- ggplot(allrep_df, aes(x = overlap)) +
  geom_histogram(color="black", fill="gray70") +
  xlab("Number of papers shared between reviews") +
  ylab("Frequency of overlap for Reporting criteria") +
  niceplot

# save figure S1

ggsave("figures/Fig_S1.pdf", hist_rep, width = 8, height = 5, dpi = 300)

# display figure S1

hist_rep
```

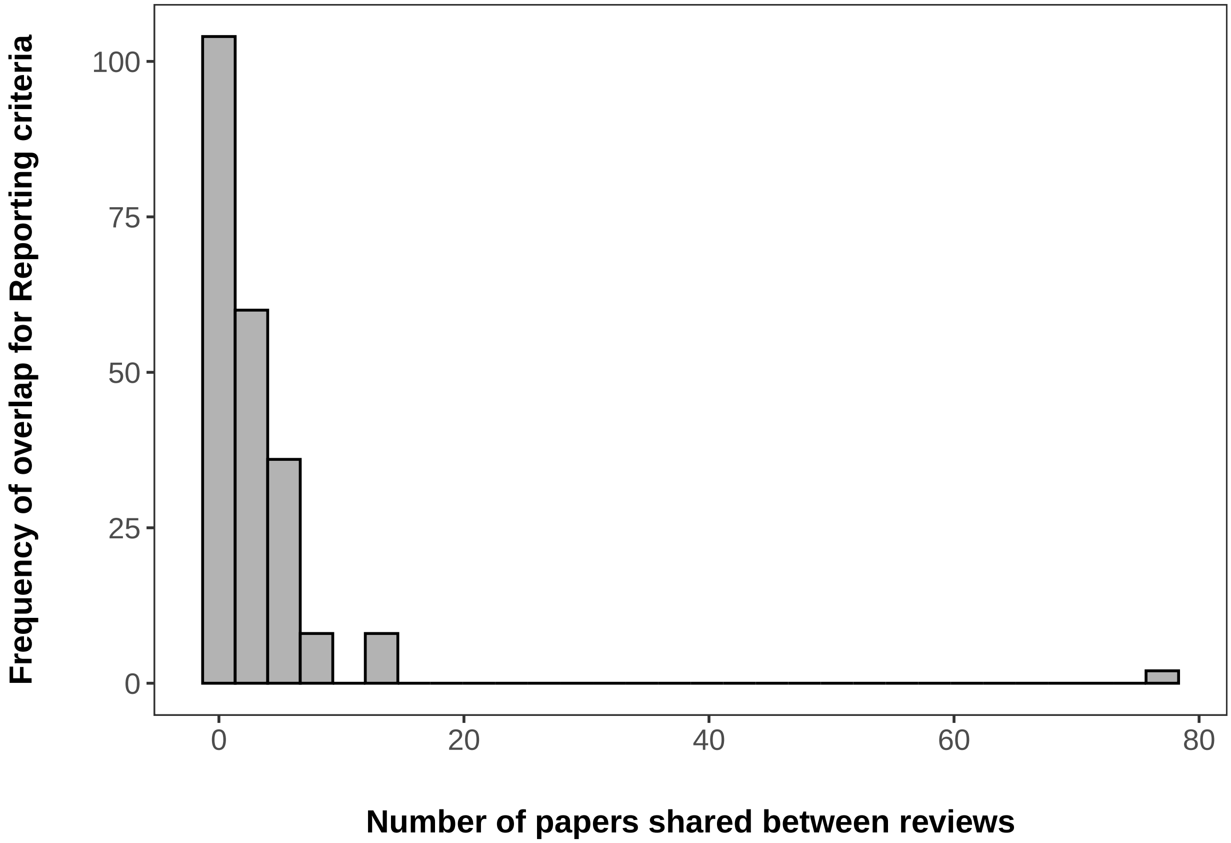

Fig S1. Distribution of paper overlap for Reporting criteria. Distribution of the number of papers shared between reviews for all the Reporting criteria combined.

Matrix of overlap for Execution criteria

Weighted effect sizes by study precision:

```
# calculate number of papers shared by reviews
ec1 <- getPercentOverlapMatrix(toplot, criterion = exevec[1], vecs = vecs)
ec1
```

```
            arch2015 kori2014 papp2020 phil2012 vett2013 lodi2021_fe
arch2015          18        2        0        0        0           0
kori2014           2      322        3        5       30           5
papp2020           0        3       96        0        0           3
phil2012           0        5        0       55        9           0
vett2013           0       30        0        9       83           4
lodi2021_fe        0        5        3        0        4         114
lodi2021_ee        2        5        3        0        7           1
beil2022           0        6       13        6        3           0
roma2021a          0        9        2        0        2           2
            lodi2021_ee beil2022 roma2021a
arch2015              2        0         0
kori2014              5        6         9
papp2020              3       13         2
phil2012              0        6         0
vett2013              7        3         2
lodi2021_fe           1        0         2
lodi2021_ee          86        1         0
beil2022              1      217         7
roma2021a             0        7        65
```

```
# extract overlap elements
ec1_o <- ec1[lower.tri(ec1)]
```

Quantified heterogeneity in effect sizes:

```
# calculate number of papers shared by reviews
ec2 <- getPercentOverlapMatrix(toplot, criterion = exevec[2], vecs = vecs)
ec2
```

```
            arch2015 gate2002 kori2014 odea2021 seni2016 vett2013 lodi2021_fe
arch2015          18        0        2        0        3        0           0
gate2002           0       29        2        0        5        0           4
kori2014           2        2      322        5       77       30           5
odea2021           0        0        5      101       12        2           2
seni2016           3        5       77       12      325       32           8
vett2013           0        0       30        2       32       83           4
lodi2021_fe        0        4        5        2        8        4         114
beil2022           0        0        6        1        7        3           0
roma2021a          0        0        9        0        5        2           2
            beil2022 roma2021a
arch2015           0         0
gate2002           0         0
kori2014           6         9
odea2021           1         0
```

```
seni2016           7          5
vett2013           3          2
lodi2021_fe        0          2
beil2022         217          7
roma2021a          7         65
```

```r
# extract overlap elements
ec2_o <- ec2[lower.tri(ec2)]
```

Explored causes of heterogeneity:

```r
# calculate number of papers shared by reviews
ec3 <- getPercentOverlapMatrix(toplot, criterion = exevec[3], vecs = vecs)
ec3
```

```
            arch2015 gate2002 kori2014 odea2021 phil2012 robe2006 seni2016
arch2015          18        0        2        0        0        0        3
gate2002           0       29        2        0        0        0        5
kori2014           2        2      322        5        5        2       77
odea2021           0        0        5      101        0        0       12
phil2012           0        0        5        0       55        0        6
robe2006           0        0        2        0        0       73        2
seni2016           3        5       77       12        6        2      325
vett2013           0        0       30        2        9        2       32
lodi2021_fe        0        4        5        2        0        0        8
beil2022           0        0        6        1        6        0        7
            vett2013 lodi2021_fe beil2022
arch2015           0           0        0
gate2002           0           4        0
kori2014          30           5        6
odea2021           2           2        1
phil2012           9           0        6
robe2006           2           0        0
seni2016          32           8        7
vett2013          83           4        3
lodi2021_fe        4         114        0
beil2022           3           0      217
```

```r
# extract overlap elements
ec3_o <- ec3[lower.tri(ec3)]
```

Tested for publication bias:

```r
# calculate number of papers shared by reviews
ec4 <- getPercentOverlapMatrix(toplot, criterion = exevec[4], vecs = vecs)
ec4
```

```
            arch2015 gate2002 kori2014 odea2021 papp2020 phil2012 robe2006
arch2015          18        0        2        0        0        0        0
gate2002           0       29        2        0        0        0        0
kori2014           2        2      322        5        3        5        2
odea2021           0        0        5      101        3        0        0
```

```
papp2020           0        0        3        3       96        0        0
phil2012           0        0        5        0        0       55        0
robe2006           0        0        2        0        0        0       73
lodi2021_fe        0        4        5        2        3        0        0
lodi2021_ee        2        4        5        4        3        0        1
beil2022           0        0        6        1       13        6        0
roma2021a          0        0        9        0        2        0        0
            lodi2021_fe lodi2021_ee beil2022 roma2021a
arch2015              0           2        0         0
gate2002              4           4        0         0
kori2014              5           5        6         9
odea2021              2           4        1         0
papp2020              3           3       13         2
phil2012              0           0        6         0
robe2006              0           1        0         0
lodi2021_fe         114           1        0         2
lodi2021_ee           1          86        1         0
beil2022              0           1      217         7
roma2021a             2           0        7        65
```

```
# extract overlap elements
ec4_o <- ec4[lower.tri(ec4)]
```

Conducted sensitivity analysis:

```
# calculate number of papers shared by reviews
ec5 <- getPercentOverlapMatrix(toplot, criterion = exevec[5], vecs = vecs)
ec5
```

```
            arch2015 gate2002 kori2014 odea2021 papp2020 phil2012 robe2006
arch2015          18        0        2        0        0        0        0
gate2002           0       29        2        0        0        0        0
kori2014           2        2      322        5        3        5        2
odea2021           0        0        5      101        3        0        0
papp2020           0        0        3        3       96        0        0
phil2012           0        0        5        0        0       55        0
robe2006           0        0        2        0        0        0       73
lodi2021_fe        0        4        5        2        3        0        0
lodi2021_ee        2        4        5        4        3        0        1
            lodi2021_fe lodi2021_ee
arch2015              0           2
gate2002              4           4
kori2014              5           5
odea2021              2           4
papp2020              3           3
phil2012              0           0
robe2006              0           1
lodi2021_fe         114           1
lodi2021_ee           1          86
```

```
# extract overlap elements
ec5_o <- ec5[lower.tri(ec5)]
```

Controlled for phylogenetic non-independence:

```
# calculate number of papers shared by reviews
ec6 <- getPercentOverlapMatrix(toplot, criterion = exevec[6], vecs = vecs)
ec6
```

```
             cado2012 cham2012 jenn2012 kori2014 papp2020 lodi2021_fe
cado2012          240       20        9       74        0          13
cham2012           20       56       11       13        0           2
jenn2012            9       11       94        0        0           0
kori2014           74       13        0      322        3           5
papp2020            0        0        0        3       96           3
lodi2021_fe        13        2        0        5        3         114
lodi2021_ee        12        2       16        5        3           1
             lodi2021_ee
cado2012              12
cham2012               2
jenn2012              16
kori2014               5
papp2020               3
lodi2021_fe            1
lodi2021_ee           86
```

```
# extract overlap elements
ec6_o <- ec6[lower.tri(ec6)]
```

Multifactorial analysis of moderators:

```
# calculate number of papers shared by reviews
ec7 <- getPercentOverlapMatrix(toplot, criterion = exevec[7], vecs = vecs)
ec7
```

```
             kori2014 seni2016 lodi2021_fe
kori2014          322       77           5
seni2016           77      325           8
lodi2021_fe         5        8         114
```

```
# extract overlap elements
ec7_o <- ec7[lower.tri(ec7)]
```

Explored temporal changes in effect size:

```
# calculate number of papers shared by reviews
ec8 <- getPercentOverlapMatrix(toplot, criterion = exevec[8], vecs = vecs)
ec8
```

```
             kori2014 papp2020 lodi2021_fe lodi2021_ee
kori2014          322        3           5           5
papp2020            3       96           3           3
lodi2021_fe         5        3         114           1
lodi2021_ee         5        3           1          86
```

```r
# extract overlap elements
ec8_o <- ec8[lower.tri(ec8)]
```

This is the summary of the vector including all the number of papers shared for all Execution criteria:

```r
allex <- c(ec1_o, ec2_o, ec3_o, ec4_o, ec5_o, ec6_o, ec7_o, ec8_o)

summary(allex)
```

```
   Min. 1st Qu.  Median    Mean 3rd Qu.    Max.
  0.000   0.000   2.000   4.487   5.000  77.000
```

This is the distribution of overlap for all Execution criteria combined:

```r
# create dataframe to plot with ggplot

allex_df <- data.frame(overlap = allex)

# make histogram

hist_exe <- ggplot(allex_df, aes(x = overlap)) +
  geom_histogram(color="black", fill="gray70") +
  xlab("Number of papers shared between reviews") +
  ylab("Frequency of overlap for Execution criteria") +
  niceplot

# save figure S2

ggsave("figures/Fig_S2.pdf", hist_exe, width = 8, height = 5, dpi = 300)

# display figure S3
hist_exe
```

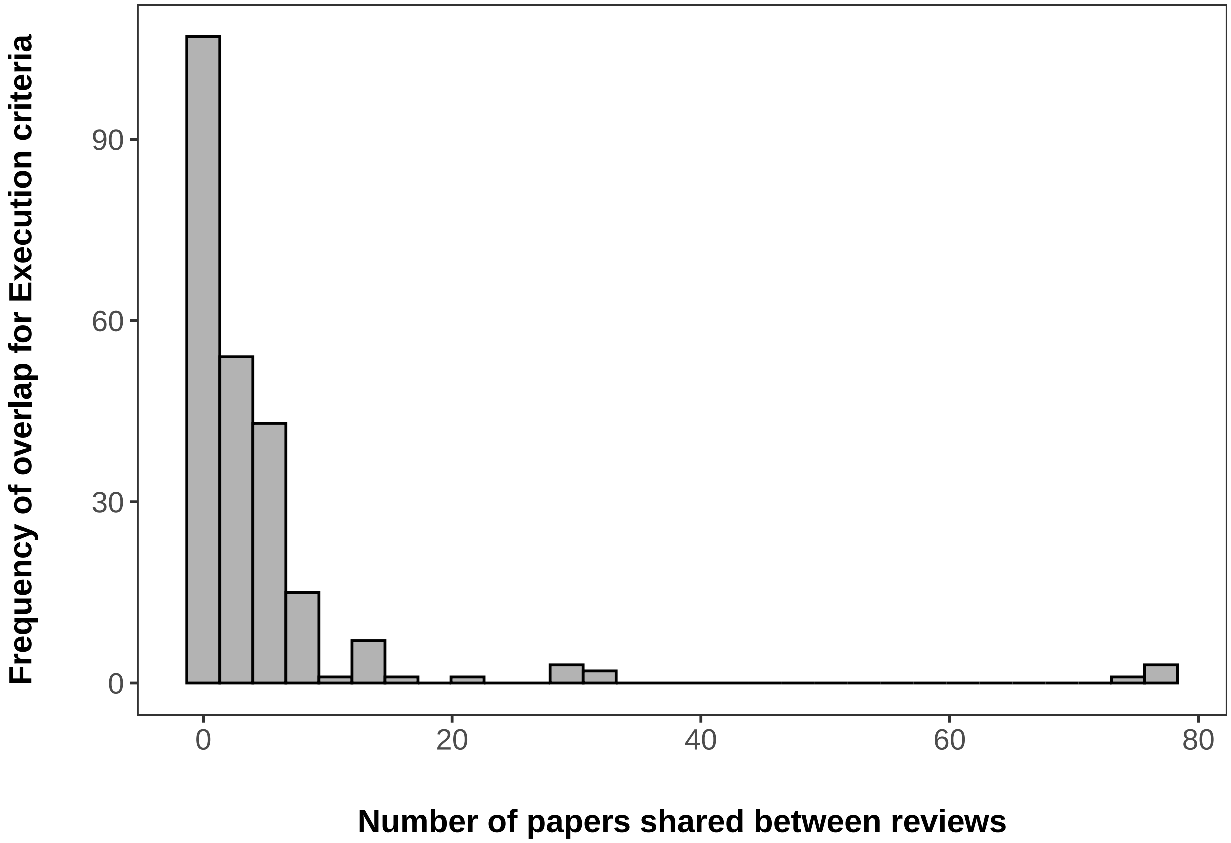

Fig S2. Distribution of paper overlap for Execution criteria. Distribution of the number of papers shared between reviews for all the Execution criteria combined.
