# Supplementary material for: A meta-evaluation of the quality of reporting and execution in ecological meta-analyses
Source: PLoS One. 2023 Oct 12;18(10):e0292606. doi: 10.1371/journal.pone.0292606 (PMC10569516; doi:10.1371/journal.pone.0292606)
Supplement: S3 Appendix — For data from Pappalardo et al. [1], we indicated when their data was re-analyzed or when we collected new data in this study by re-reviewing their compilation of ecological meta-analyses with the tag “added”. When the number of publications complying (or not complying) with one of the criteria was reported, we used that information to calculate the percentage of papers complying; in other cases the reviews directly reported the information as a percentage. In a few papers in which we had the original review data for each criterion [e.g., 18], we summed the number of papers complying with each criterion, and then calculated the percentage of compliance based on the total number of papers relevant for that criterion. (PDF) [file pone.0292606.s010.pdf]

Appendix S3

Paula Pappalardo, Chao Song, Bruce A. Hungate, Craig W. Osenberg

From: A meta-evaluation of the quality of reporting and execution in ecological meta-analyses

Details on the information extracted from each review paper for each performance criterion

For data from Pappalardo et al. [1], we indicated when their data was re-analyzed or when we collected new data in this study by re-reviewing their compilation of ecological meta-analyses with the tag "added". When the number of publications complying (or not complying) with one of the criteria was reported, we used that information to calculate the percentage of papers complying; in other cases, the reviews directly reported the information as a percentage. In a few papers in which we had the original review data for each criterion [e.g., 18], we summed the number of papers complying with each criterion, and then calculated the percentage of compliance based on the total number of papers relevant for that criterion.

Criterion/publication	Details
REPORTING	
Full details of bibliographic searches	
Archmiller et al. [18]	The authors evaluated separately if studies reported the "Boolean operators" and "Search databases and dates", we used both columns since they presented the raw data. In their Table S1, we counted as 1 when there was compliance for both items and calculated the percentage dividing by the total number of meta-analyses reviewed.
Beillouin et al. [19]	Data extracted from criterion "Search strings are clearly presented". Percentage calculated from the column "Quality_Lit_strings_OK" in the supplementary data package, n = 217 meta-analyses) and verified when possible with the main text and Figure 5.
Gates [10]	Data extracted from criterion "methods used to locate primary studies". We acknowledge that their concept is less strict, because compliance was considered as long as the study reported the source of the information but did not assess reproducibility.

Koricheva & Gurevitch [7]	Data extracted from criterion “Are details of bibliographic search (electronic data bases used, keyword combinations, years) reported in sufficient detail to allow replication?”, Table 3.
Lodi et al. [17]	Data extracted from criterion “Searching Details” in Tables 3 and 4. Detailed description of each criteria appears in their Table 2. The authors followed Koricheva & Gurevitch [7] list of criteria.
Philibert et al. [25]	Data extracted from the main text for criterion “Repeatable procedure: A repeatable procedure for the selection of papers for the meta-analysis is presented.”
ODea et al. [2]	Data extracted from criterion 5.4 of the PRISMA EcoEvo checklist (Table 1): “Provide enough information to repeat the equivalent search (if possible), including the timespan covered (start and end dates)”.
Pappalardo et al. [1]	Reanalyzed from Pappalardo et al [1] dataset looknig at information from two columns. We assigned 1 when columns *lit.search.explained* and *keywords.explained* were “yes”. The rest of the studies were coded as 0. The paper that used their own data for the meta-analysis was considered as not relevant.
Roberts et al. [26]	Data extracted from criterion "Defined search terms to be used to identify sources of evidence", Table 1.
Romanelli et al. [16]	Matched to the “2.2 Search strings clearly defined” criteria column in their supplementary data file. We only consider the 63 meta-analyses and did not included the systematic reviews. We assigned 0 to their score 0, 0.5 points to their score for partial compliance (1), and 1 point to their score of full compliance (3). We calculated percent compliance as the (sum of points*100)/total meta-analyses reviewed.
Inclusion/exclusion criteria	
Archmiller et al. [18]	Counted “1”s in Table S1 for item “Exclusion/inclusion criteria” and divided by number of articles to calculate the percentage of compliance. The item was described “Gave specific information about why papers were retained or rejected”.
Gates [10]	We extracted from the main text the number of studies that reported an explicit criterion for inclusion of studies, and use it to calculate the percentage of compliance.
Koricheva & Gurevitch [7]	Data extracted from criterion “Reporting inclusion/exclusion criteria”, Table 3.
Lodi et al. [17]	Data extracted from criterion “Inclusion/exclusion” in Tables 3 and 4. Detailed description of each criteria appears in Table 2. The authors followed Koricheva & Gurevitch [7] list of criteria.
ODea et al. [2]	Data extracted from criterion 4.1 of the PRISMA EcoEvo checklist (Table 1): “Report the specific criteria used for including or excluding studies when screening titles and/or abstracts, and full texts, according to the aims of the systematic review (e.g. study design, taxa, data availability)”.
Pappalardo et al. [1] (added)	Each study was coded 1 if the authors explained the inclusion/exclusion criteria to determine which papers to include in their meta-analysis. We gave 0.5 points to studies in which this was partially explained. The study that used their own data was tagged as “not applicable” and it was filtered out to calculate the percentage of studies complying with this criterion.

Roberts et al. [26]	Data extracted from criterion "Defined inclusion/exclusion criteria for identification of relevant (evidence) studies", Table 1.
Romanelli et al. [16]	Matched to the "3.1 Inclusion criteria documented for all studies" criteria column in their supplementary data file. We only consider the 63 meta-analyses, and did not included the systematic reviews. We assigned 0 to their score 0, 0.5 points to their score for partial compliance (1), and 1 point to their score of full compliance (3). We calculated percent compliance as the (sum of points*100)/total meta-analyses reviewed.
Reference list of primary studies	
Archmiller et al. [18]	Data extracted from criterion "List of References".
Gates [10]	Data extracted from number of papers that "gave lists of studies that were included".
Koricheva & Gurevitch [7]	Data extracted from criterion "Have full bibliographic details of primary studies included in a meta-analysis been provided?"
Lodi et al. [17]	Data extracted from criterion "Bilbiographic details" in Tables 3 and 4. Detailed description of each criteria appears in Table 2. The authors followed Koricheva & Gurevitch [7] list of criteria.
ODea et al. [2]]	Data extracted from criterion 27.1 of the PRISMA EcoEvo checklist (Table 1): "References".
Pappalardo et al. [1]	We assigned 1 to each study that provided the full references (column *ref.provided* indicating "yes"); we assigned 0.5 to the few studies in which only partial information was provided (e.g., only providing first author and year or only providing references for one of the analysis conducted in the paper). The paper that used their own data for the meta-analysis was considered as not relevant.
Philibert et al. [25]	Data extracted from criterion "References: a list of the references used for the meta-analysis is provided."
Roberts et al. [26]	Data extracted from criterion "Provides references of all studies within the review".
Meta-analytical model	
Archmiller et al. [18]	Counted number of "1"s for "Model choice" in their Table S1. Item was described as "Stated if fixed-effect or random-effects model was used or discussed between- and within-study variation".
Beillouin et al. [19]	Data extracted from criterion "Statistical models are fully described"; percentage calculated from the data available in the supplementary data package, column "Quality_Model" (n = 217 meta-analyses).
Koricheva & Gurevitch [7]	Data extracted from criterion "Specifying the meta-analytical model", Table 3.
Lodi et al. [17]	Data extracted from criterion "Meta-analytical model" in Tables 3 and 4. Detailed description of each criteria appears in Table 2. The authors followed Koricheva & Gurevitch [7] list of criteria.
ODea et al. [2]	Data extracted from criterion 12.1 of the PRISMA EcoEvo checklist (Table 1): "Describe the models used for synthesis of effect sizes".

Pappalardo et al. [1]	The cases in which the column *data.analysis* indicated "not mentioned", or "not clear", were coded 0. When the model was reported it was coded 1. For the few cases in which a non-traditional meta-analytic model was used to analyze the effect sizes, but it was explained, we also coded it as 1.
Senior et al. [27]	Percentage of papers that did not report the meta-analytic model used was extracted from the text.
Dataset used in the meta-analysis	
Archmiller et al. [18]	Data extracted from criterion "Dataset used in meta-analysis", detailed as "Provided data set used for meta-analysis in article, SI, or online". We counted the number of "1s" and divided it by the number of studies to obtain the percentage.
Koricheva & Gurevitch [7]	Data extracted from criterion "Has the data set used for meta-analysis, including effect sizes and variances/sample sizes from individual primary studies and moderator variables, been provided as electronic appendix?".
Lodi et al. [17]	Data extracted from criterion "Data" in Tables 3 and 4. Detailed description of each criteria appears in Table 2. The authors followed Koricheva & Gurevitch [7] list of criteria.
ODea et al. [2]	Data extracted from criterion 18.2 in the PRISMA EcoEvo checklist (Table 1): "Share data required to reproduce the results presented in the manuscript".
Pappalardo et al. [1]	We coded 1 the studies with a "yes" in the column *original.data.provided*; we also coded 1 studies with a "yes" in both the column *eff.provided* and *var.provided*. We coded 0 studies where no data was provided, only partial data was provided, or those few cases in which the appendix links did not work and the authors did not reply to the emails requesting the original dataset.
Philibert et al. [25]	Data extracted from criterion "Availability of the dataset" item, described as "The dataset is available in an electronic format or published directly in the paper".
Data used to calculate effect sizes (raw data)	
Beillouin et al. [19]	Data extracted from criterion "The full dataset is available" (also detailed in text as "data of the original studies"); percentage calculated from the data available in the supplementary data package, column "Quality_Data_Sharing" (n = 217 meta-analyses).
Pappalardo et al. [1]	Data extracted from column *original.data.provided*, coding 1 the studies with a "yes"; 0.5 the studies with a "partially", and 0 studies with a "no" (or those few cases in which the appendix links did not work and the authors did not reply to the emails requesting the original dataset).
Romanelli et al. [16]	Data extracted from criterion "5.2 Are the extracted data reported?" in their supplementary data file. We only consider the 63 meta-analyses, and did not included the systematic reviews. We assigned 0 to their score 0, 0.5 points to their score for partial compliance (1), and 1 point to their score of full compliance (3). We calculated percent compliance as the (sum of points*100)/total meta-analyses reviewed.
The number of studies and effect sizes	

Cadotte et al. [8]	Data extracted from the Supplementary Material 2. To calculate percent compliance we coded 1 the cases in which there was information for the two columns *No_publications* and *No_datasets_or_studies*.
ODea et al. [2]	Data extracted from criterion 20.1 of the PRISMA EcoEvo checklist (Table 1): "Report the number of studies and effect sizes for data included in meta-analyses".
Pappalardo et al. [1] (added)	We collected information on the number of effect sizes reported in each meta-analysis from the main text if available, from the supplementary material, or by counting rows in the supplementary data tables. We combined this information with the number of studies included in the meta-analysis reported by Pappalardo et al. [1] and if both were reported we coded the item "Report the number of studies and effect sizes" as 1. We corrected one mistake in the dataset by Pappalardo et al. [1]; The publication by Luo et al. (2015) was reported as having included 216 papers, but that was before excluding some papers, the correct final number of papers should have been 31, which is the one we used in this paper.
The software used	
Archmiller et al. [18]	We counted number of "1"s for "Statistical software used" in Table S1 and divided it by the number of studies to obtain the percentage of compliance.
Koricheva & Gurevitch [7]	Data extracted from criterion "Specifying the software used", Table 3.
Lodi et al. [17]	Data extracted from criterion "Software" in Tables 3 and 4. Detailed description of each criteria appears in Table 2. The authors followed Koricheva & Gurevitch [7] list of criteria.
Nakagawa & Santos [23]	The Appendix reported the number of papers that did not report the software used, that we used to calculate the percentage of compliance.
ODea et al. [2]	Data extracted from criterion 13.1 of the PRISMA EcoEvo checklist (Table 1): "Describe the statistical platform used for inference (e.g. R)".
Pappalardo et al. [1]	The cases in which the column *software* indicated "not mentioned" were coded as 0, and cases in which any software was reported coded as 1.
Philibert et al. [25]	Data extracted from Table 3 that reported the software used in the meta-analysis to calculate percentage of papers reporting the software. Note that Philibert evaluated if the software "was made available" which is more stringent criteria than generally reporting the software.
Senior et al. [27]	Number or papers reporting the software were extracted from the "Data_Package_Part_3_Survey_Data" excel spreadsheet.
The packages used (if applicable)	
ODea et al. [2]	Data extracted from criterion 13.2 of the PRISMA EcoEvo checklist (Table 1): "Describe the packages used to run models".
Pappalardo et al. [1] (added)	When papers used a programming language, we coded this criterion 1 if they described the packages used.
The functions used (if applicable)	
ODea et al. [2]	Data extracted from criterion 13.3 of the PRISMA EcoEvo checklist (Table 1): "Describe the functions used to run models".

Pappalardo et al. [1] (added)	When papers used a programming language, we coded this criterion 1 if they described the functions used.
The code (if applicable)	
ODea et al. [2]	Data extracted from criterion 18.4 of the PRISMA EcoEvo checklist (Table 1): “Share analysis scripts”.
Pappalardo et al. [1] added	When papers used a programming language, we coded this criterion 1 if they provided the code.
The types of non-independence	
Archmiller et al. [18]	Data extracted from criterion “Quantified or stated impacts of nonindependence”.
Lodi et al. [17]	Data extracted from criterion “Multiple effect sizes”, explained as “If more than one estimate of effect size per study was included in the analysis, has potential non-independence of these estimates been considered?”.
ODea et al. [2]	Data extracted from criterion 14.1 of the PRISMA EcoEvo checklist (Table 1): “Describe the types of non-independence encountered (e.g. phylogenetic, spatial, multiple measurements over time)”.
Pappalardo et al. [1] (added)	Each study was coded 1 if the authors discussed instances of non-independence in their data. Common cases of non-independence mentioned were repeated measures, multiple data per study, multiple experimental levels, multiple data per species.
EXECUTION	
Weighed effect sizes by study precision	
Archmiller et al. [18]	We counted the number of "1"s in their item “Individual study weight calculation” in Table S1 and divided it by the number of studies to obtain the percentage of compliance.
Beillouin et al. [19]	Data extracted from criterion “Studies are weighted according to their accuracy”; percentage calculated from the data available in the supplementary data package, column “Quality_Model_weights” (n = 217 meta-analyses).
Koricheva & Gurevitch [7]	Data extracted from criterion “Have effect sizes been weighted by study precision or has the rational for using unweighted approach been provided?”
Lodi et al. [17]	Data extracted from criterion “Weighted effect sizes” in Tables 3 and 4. Detailed description of each criteria appears in Table 2. The authors followed Koricheva & Gurevitch [7] list of criteria.
Pappalardo et al. [1]	To quantify if a study weighted the effect sizes we first re-classified all the levels of the column *weighting* into “yes”, “no”, or “not mentioned”. To calculate the percent of studies that weighted their effect sizes, we assigned 1 to studies with “yes”, and 0 to studies that did not weight or that did not mention if they used weights. The study that conducted both weighted and unweighted analysis and only reported unweighted was coded as 1. We also quantified separately the studies that used more traditional meta-analysis weights (sample size, inverse variance).
Philibert et al. [25]	Data extracted from criterion “Weighting: Observations are weighted according to their level of accuracy in the statistical model”.

Romanelli et al. [16]	Data extracted from criterion "4.2 Studies were weighted according to the methodological quality?" in their supplementary data file. We only consider the 63 meta-analyses, and did not included the systematic reviews. In this case, we assigned 0 to their score 0 and 1 (if weighted was not transparent or removed studies), and only assigned 1 to their score of 3 (metric for weighting was clearly stated). We calculated percent compliance as the (sum of points*100)/total meta-analyses reviewed.
Vetter et al. [28]	The total from this percentage is from the 60 articles that fulfilled all requirements plus the 23 that fulfilled all but weighting (total = 83). So, the 37 that did weight represent a 44%. Information extracted from the text.
Tested for publication bias	
Archmiller et al. [18]	Counted "1"s in Table S1 for item "Publication Bias" and divided by number of articles to calculate the percentage of compliance. The item was described as "Discussed or quantified publication bias".
Beillouin et al. [19]	Data extracted from criterion "Publication bias is analyzed"; percentage calculated from the data available in the supplementary data package, column "Quality_Publication_bias" (n = 217 meta-analyses).
Gates [10]	The number of papers that reported to discussed publication bias and that calculated the "fail-safe" number was extracted from the text. Percentage calculated based on the total numbers of studies analyzed.
Koricheva & Gurevitch [7]	Data extracted from criterion "Testing for publication bias", Table 3.
Lodi et al. [17]	Data extracted from criterion "Publication bias" in Tables 3 and 4. Detailed description of each criteria appears in Table 2. The authors followed Koricheva & Gurevitch [7] list of criteria.
Nakagawa & Santos [23]	The percentage of papers that utilized some kind of procedure was extracted from the main text.
ODea et al. [2]	Data extracted from criterion 24.1 of the PRISMA EcoEvo checklist (Table 1): "Provide results for the assessments of the risks of bias (e.g. Egger's regression, funnel plots)".
Pappalardo et al. [1] (added)	Each study was coded 1 if the authors addressed publication bias (usually this was done using funnel plots or the fail-safe number); the study that used their own data was filtered out to calculate the percentage of studies complying with this criterion.
Philibert et al. [25]	Data extracted from criterion "Investigation of publication bias", described as "Assessment of the publication bias, which occurs when only studies with highly significant results are published. In this case, a meta-analysis can lead to a biased conclusion and an overestimation of the effect of a given factor. Publication bias is a predominant issue in meta-analysis and several methods such as funnel plots (e.g., Borenstein et al. 2009; Light and Pillemer, 1984) have been developed to detect the presence of such bias in datasets including published results."
Roberts et al. [26]	Data extracted from criterion "Estimation of publication bias", Table 1.
Romanelli et al. [16]	Data extracted from criterion "6.3 Does the synthesis consider possible publication bias?" criteria column in their supplementary data file. We only consider the 63 meta-analyses, and did not included the systematic reviews. For this criterion, we consider any way of addressing publication bias as complying, since that seems to be how other papers have measured it (including our data extraction for meta-analyses from Pappalardo et al. [1]). So we assigned 0 to their score 0, and 1 to their scores for partial (1) and full (3) compliance. We calculated percent compliance as the (sum of points*100)/total meta-analyses reviewed.
Conducted sensitivity analysis	

Archmiller et al. [18]	Counted “1”s in Table S1 for item “Sensitivity Analysis” and divided by number of articles to calculate the percentage of compliance. The item was described as “Quantified impact of individual effect sizes with sensitivity analysis or discussed potential impacts of individual study effect sizes”.
Gates [10]	The number of papers that perform a sensitivity analysis was extracted from the text. Percentage calculated based on the total numbers of studies analyzed.
Koricheva & Gurevitch [7]	Data extracted from criterion “Sensitivity analysis”, Table 3.
Lodi et al. [17]	Data extracted from criterion “Sensitivity analysis” in Tables 3 and 4. Detailed description of each criteria appears in Table 2. The authors followed Koricheva & Gurevitch [7] list of criteria.
ODea et al. [2]	Data extracted from criterion 24.2 of the PRISMA EcoEvo checklist (Table 1): “Provide results for the robustness of the review’s results (e.g. subgroup analyses, meta-regression of study quality, results from alternative methods of analysis, and temporal trends)”.
Pappalardo et al. [1] (added)	Each study was coded 1 if the authors conducted some type of sensitivity analysis, such as comparing their results with or without influential points, comparing weighted and weighted analysis, or comparing different types of effect size measures.
Philibert et al. [25]	Data extracted from criterion “Sensitivity analysis”, described as “Analysis of the sensitivity of the conclusions to any change in the dataset and/or in the statistical method used to analyze the data. Sensitivity analyses should be carried out to identify influential data and to assess the robustness of the main conclusions of a meta-analysis to the assumptions made in the statistical analysis.”
Roberts et al. [26]	Data extracted from criterion “Sensitivity analysis”, Table 1.
Controlled for phylogenetic non-independence	
Cadotte et al. [8]	Data was extracted from the data table presented in the Supplementary Material, using additional metadata provided by Marc Cadotte. If the column *No_species* was higher than 3 (following the methods in Chamberlain et al. [20]), we quantified if studies corrected for phylogenetic non-independence using the column *Phylogeny_included*. To calculate the percent compliance, we coded as 1 studies where Cadotte et al. [8] reported the meta-analysis used a real phylogeny (*Phylogeny_included == 1)*, and as 0.5 when it was reported that the meta-analysis was for congeneric species (*Phylogeny_included == 2)*, or that it used taxonomic levels (*Phylogeny_included == 3*). The 4 studies that had a large number of species and Phylogeny_included was empty were coded 0.
Chamberlain et al. [20]	Information extracted from figure 1, only 2 papers performed a phylogenetic meta-analysis (coded as 1 for full compliance), and 19 assessed wether effect sizes differed among taxonomic categories (coded as 0.5 for partial compliance), 35 did traditional meta-analysis (coded as zero). We calculated the percentage of compliance from the total number of relevant papers that were analyzed (n = 56).
Jennions et al. [22]	From their Table 1, we extracted the number of papers that controlled for phylogeny, the number of papers that did not, and the number of papers for which it was not relevant. We calculated the percentage of compliance from the total number of relevant papers that were analyzed.
Koricheva & Gurevitch [7]	Data extracted from criterion “Controlling for phylogeny”, Table 3.
Lodi et al. [17]	Matched to the “Controlling for phylogeny” criterion in Tables 3 and 4. Detailed description of each criteria appears in Table 2. The authors followed Koricheva & Gurevitch [7] list of criteria.

Nakagawa & Santos [23]	From their Appendix, we extracted the percentage of papers that did not control for phylogenetic relatedness, and transform it into percentage of compliance using the total number of papers analyzed.
Pappalardo et al. [1] (added)	Each study was coded as 1 if the authors corrected the analysis for phylogenetic relatedness (when applicable), and was coded 0.5 if phylogenetic effects were partially addressed (e.g., by using family or taxonomy as a covariate). Community or ecosystem studies were coded "not applicable" and filtered out before calculating the percentage of papers complying with this criterion.
Quantifying heterogeneity in effect sizes	
Archmiller et al. [18]	Counted "1"s in Table S1 for item "Heterogeneity estimation" and divided by number of articles to calculate the percentage of compliance. The item was described as "Calculated heterogeneity statistics (e.g. Q, s, I)".
Beillouin et al. [19]	Heterogeneity data was combined in their category "Heterogenity of results is analyzed"; but we were able to calculate the percentage that quantified heterogeneity by counting the level "YES, quantification (I2, Qb) and subgroup analysis" in the column "Quality_Model_heterogeneity (I2, PI, …)" available from the supplementary data package spreadsheet (n = 217 meta-analyses).
Gates [10]	We extracted from the text the number of studies that "contained some exploration of the heterogeneity of results, using the Q statistic or another measure of homogeneity of effect sizes.
Koricheva & Gurevitch [7]	Data extracted from criterion "Quantifying heterogeneity in effect sizes", Table 3.
Lodi et al. [17]	Data extracted from criterion "Heterogeneity in effect sizes" in Table 3. Detailed description of each criteria appears in Table 2. The authors followed Koricheva & Gurevitch [7] list of criteria.
ODea et al. [2]	Data extracted from criterion 22.1 of the PRISMA EcoEvo checklist (Table 1): "Report indicators of heterogeneity in the estimated effect (e.g. I2, tau2 and other variance components)".
Romanelli et al. [16]	Data extracted from criterion "6.2 Is heterogeneity in the effect of the Intervention/Exposure investigated statistically?" in their supplementary data file. We only consider the 63 meta-analyses, and did not included the systematic reviews. In this case, all the studies were scored at the maximum value (3) and tallied by us as 1. We calculated percent compliance as the (sum of points*100)/total meta-analyses reviewed.
Senior et al. [27]	We extracted from the text the percentage of studies that reported statistics associated with heterogeneity.
Vetter et al. [28]	We extracted from the text the percentage of articles in which "authors quantified heterogeneity using an index measure".
Exploring causes of heterogeneity	
Archmiller et al. [18]	Counted "1"s in Table S1 for item "Sub-group analysis" and divided by number of articles to calculate the percentage of compliance. The item was described as "Summarized effect sizes for subgroups or categorical covariates".
Beillouin et al. [19]	Heterogeneity data was combined in their category "Heterogenity of results is analyzed"; but we were able to calculate the percentage that explored heterogeneity by counting both the level "YES, quantification (I2, Qb) and subgroup analysis" and "YES, analysis by moderators/subgroups" in the column "Quality_Model_heterogeneity (I2, PI, …)" available from the supplementary data package spreadsheet (n = 217 meta-analyses).

Chaudhary et al. [21]	Percentage extracted from the text for “studies that collected data on multiple predictor variables” (not to be confused with multifactor analysis that was reported separately by the authors).
Gates [10]	Number of papers that reported results for subgroups was extracted from the main text “Eighteen reviews reported results for various subgroups of the studies or individuals included. In most cases these appeared to have been prespecified, as they were natural divisions into different classes of study (for example, different trophic groups, different habitats or different environmental stresses)”. We calculated the percentage of compliance based on the numbers of papers analyzed.
Koricheva & Gurevitch [7]	Data extracted from criterion “Exploring causes of heterogeneity”,Table 3. It seems to be specific to meta-regression based on their question: “Have the causes of existent heterogeneity in effect sizes been explored by meta-regression?”.
Lodi et al. [17]	Data extracted from criterion “Causes of heterogeneity” in Table 3. Detailed description of each criteria appears in Table 2. The authors followed Koricheva & Gurevitch, 2014 list of criteria.
ODea et al. [2]	Data extracted from criterion 15.1 of the PRISMA EcoEvo checklist (Table 1): “Provide a rationale for the inclusion of moderators (covariates) that were evaluated in meta-regression models”.
Philibert et al. [25]	Information extracted from text for criterion 3, Heterogeneity was defined as "The origins of the variability of the results are analyzed", or more detailed explanation “Analysis of the variability of the results of individual studies, including checking to see whether the results vary between the selected individual studies and, when relevant, investigation of the sources of between-study variability (e.g., using random effects model).”
Roberts et al. [26]	Data extracted from criterion “Investigation of sources of heterogeneity”, Table 1, that included both “sub-group” and “meta-regression”. Data was extracted from the “sub-group” category.
Senior et al. [27]	The number of studies that reported “Analyses to identify sources of heterogeneity (e.g., meta-regression/sub-setting)” was extracted from the main text and converted to percentage of papers based on the total number of papers analyzed.
Vetter et al. [28]	The percentage of articles in which “authors explored heterogeneity by including explanatory variables” was extracted from the main text.
Multifactorial analysis of moderators	
Chaudhary et al. [21]	Number and percentage extracted from the text for studies that conducted “multifactor analysis”.
Koricheva & Gurevitch [7]	Data extracted from criterion “Multifactorial analysis of moderators”, Table 3.
Lodi et al. [17]	Matched to the “Collinearity analysis” criterion in Tables 3 and 4. Detailed description of each criteria appears in Table 2. The authors followed Koricheva & Gurevitch, 2014 list of criteria.
Senior et al. [27]	We extracted from the text the number of studies “in which several moderators are fitted in one model.
Exploring temporal changes in effect size	
Koricheva & Gurevitch [7]	Data extracted from criterion “Exploring temporal changes in effect size”, Table 3.

Lodi et al. [17]	Data extracted from criterion "Changes in effect size" in Tables 3 and 4. Detailed description of each criteria appears in Table 2. The authors followed Koricheva & Gurevitch, 2014 list of criteria.
Nakagawa et al. [24]	Data extracted from criterion "time-lag bias tests" in their Figure 2.
Pappalardo et al. [1] (added)	Each study was coded as 1 if the authors explored temporal changes in effect size, or 0 if they did not.
