# Supplementary material for: A meta-evaluation of the quality of reporting and execution in ecological meta-analyses
Source: PLoS One. 2023 Oct 12;18(10):e0292606. doi: 10.1371/journal.pone.0292606 (PMC10569516; doi:10.1371/journal.pone.0292606)
Supplement: S4 Appendix — Details and R code for additional results about compliance of quality criteria by review discipline and temporal trends in compliance. Includes the R code to generate S3–S6 Figs. (PDF) [file pone.0292606.s011.pdf]

Appendix S4

Paula Pappalardo, Chao Song, Bruce A. Hungate, Craig W. Osenberg

From: A meta-evaluation of the quality of reporting and execution in ecological meta-analyses

Setup

```
# load objects we need, generated with the R code provided

load("objects/reporting.R")
load("objects/execution.R")
load("objects/toplot.R")

# nice format for plots

niceplot <- theme_bw() +
  theme(panel.grid.major = element_blank(),
        panel.grid.minor = element_blank(),
        axis.title.x = element_text(face = "bold", size = 14,
                                    margin = margin(t = 20, r = 0, b = 0, l = 0)),
        axis.title.y = element_text(face = "bold", size = 14,
                                    margin = margin(t = 0, r = 20, b = 0, l = 0)),
        axis.text.x = element_text(size = 13),
        axis.text.y = element_text(size = 13),
        legend.text = element_text(size = 13),
        legend.title = element_text(size = 13),
        legend.position = "right")
```

Review discipline

We compared the percent compliance from the different review papers according to the research discipline. We did not observe any clear patterns among the different sub disciplines for neither the Reporting or Execution criteria:

```
safe_colorblind_palette <- c("#88CCEE", "#CC6677", "#DDCC77", "#117733", "#332288", "#AA4499",
                             "#44AA99", "#999933", "#882255", "#661100", "#6699CC", "#888888")

#scales::show_col(safe_colorblind_palette)

# create Figure S3

fig_s3 <- ggcharts::bar_chart(
```

```
     data= reporting,
     x= paper.id,
     y= percent,
     fill = topic.area,
     facet= criteria,
 ) +
  scale_fill_manual(values = safe_colorblind_palette) +
  labs(x = "Review ID", y = "Percent of papers complying with Reporting criteria") +
  niceplot +
  theme(legend.position = "bottom")

# save figure S3

ggsave("figures/Fig_S3.pdf", fig_s3, width = 17, height = 10, dpi = 300)

# display figure S3

fig_s3
```

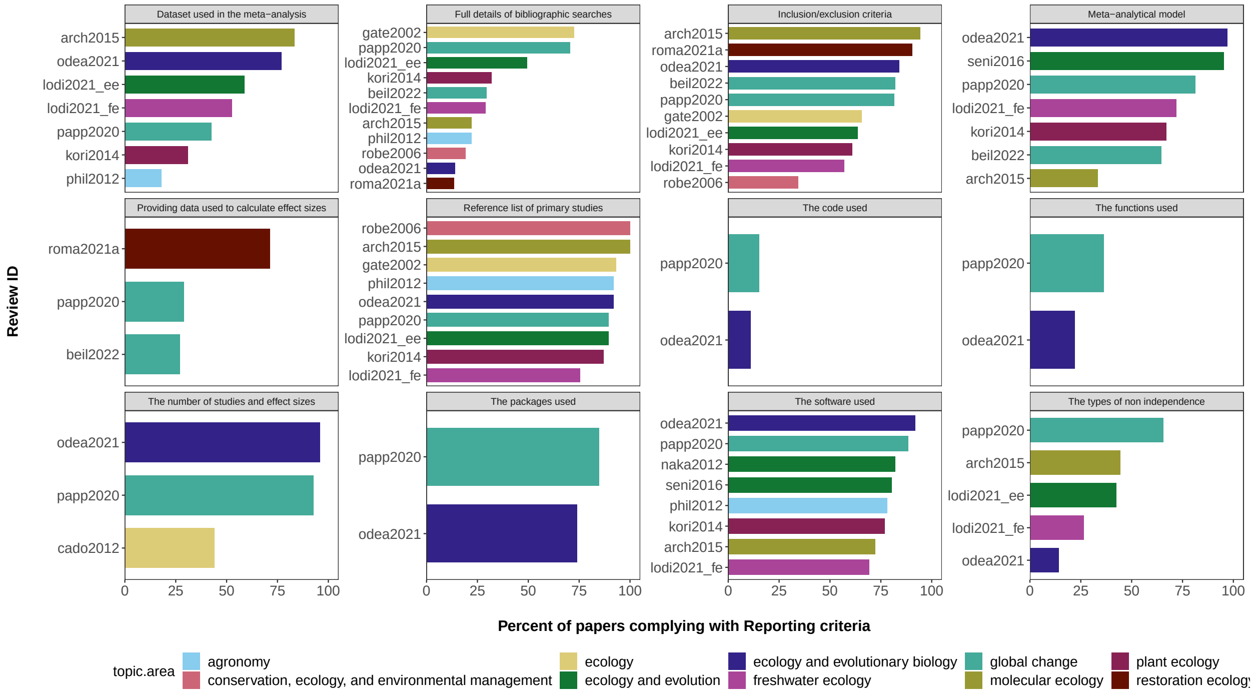

Fig S3. Percent of papers complying with Reporting criteria by review discipline. The percent of papers complying with each criterion is plotted for each synthesis paper. The colors indicate different subdisciplines of the review papers. The Review ID corresponds to the papers listed in Table 1.

```
# create Figure S4

fig_s4 <- ggcharts::bar_chart(
    data= execution,
    x= paper.id,
    y= percent,
    fill = topic.area,
```

```
    facet= criteria
  ) +
  scale_fill_manual(values = safe_colorblind_palette) +
  labs(x = "Review ID", y = "Percent of papers complying with Execution criteria") +
  ylim(0, 100)  +
  niceplot +
  theme(legend.position = "bottom")

# save figure S4

ggsave("figures/Fig_S4.pdf", fig_s4, width = 15, height = 10, dpi = 300)

# display figure S4

fig_s4
```

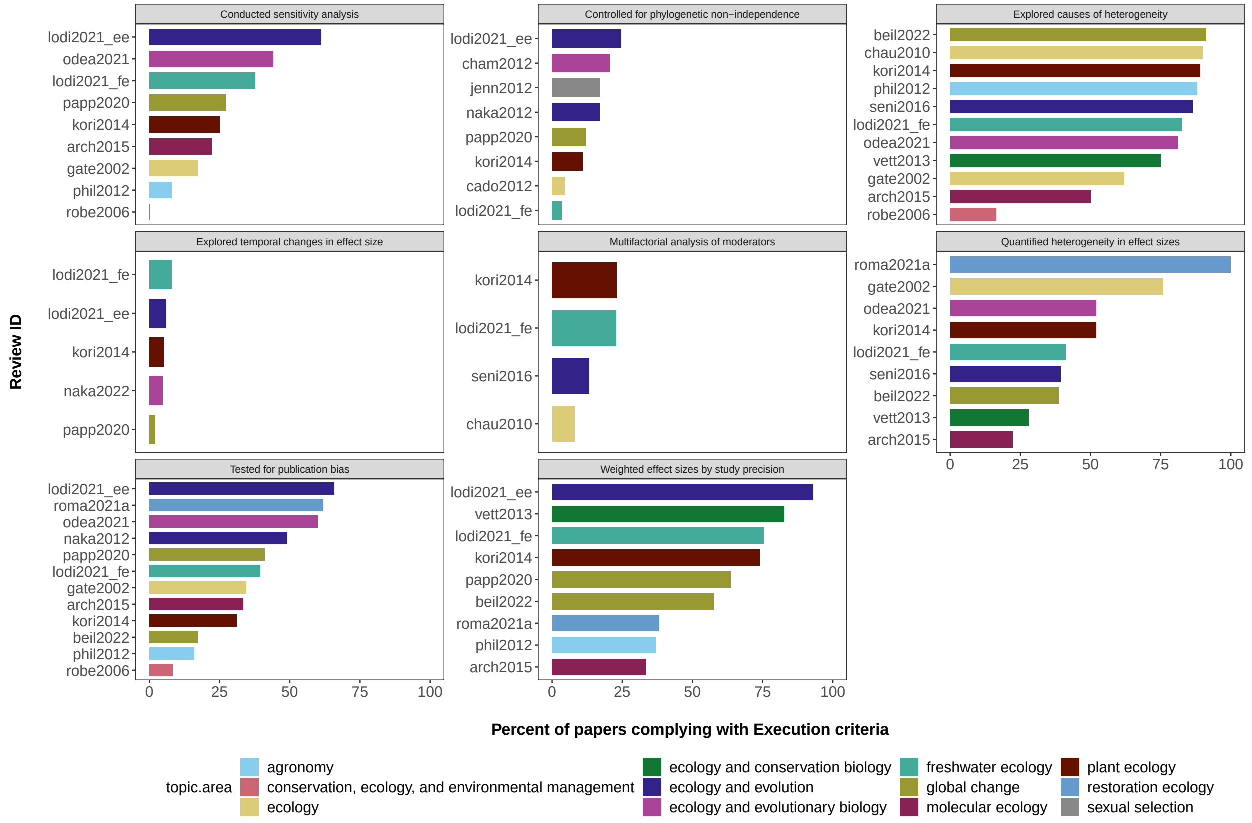

Fig S4. Percent of papers complying with Execution criteria by review discipline. The percent of papers complying with each criterion is plotted for each synthesis paper. The colors indicate different subdisciplines of the review papers. The Review ID corresponds to the papers listed in Table 1 of the main manuscript.

Temporal trends in compliance

To check for temporal trends in compliance we analyzed the time period of the papers included in each review paper and the percent compliance. We initially looked at:

1) the **mid-point** of the time period included in each review paper
2) the **earliest year** of the time period included in each review paper
3) the **most recent year** of the time period included in each review paper
4) the **full time period** included in each review paper

We considered that the **full time period** is the one that better represent the temporal data on a plot and present this below for the Reporting and Execution criteria. We did not observe any clear temporal trends.

```
# create Figure S5

fig_s5 <- ggplot(reporting, aes(mid_point, percent)) +
  geom_segment(aes(x = first_year, y = percent,
                   xend = final_year, yend = percent), data = reporting) +
  niceplot +
  facet_wrap(~criteria) +
  #ggtitle("Reporting") +
  labs(x= "Time period", y= "Percent compliance with Reporting criteria") +
  niceplot

# save figure S5

ggsave("figures/Fig_S5.pdf", fig_s5, width = 10, height = 8, dpi = 300)

# display figure S5

fig_s5
```

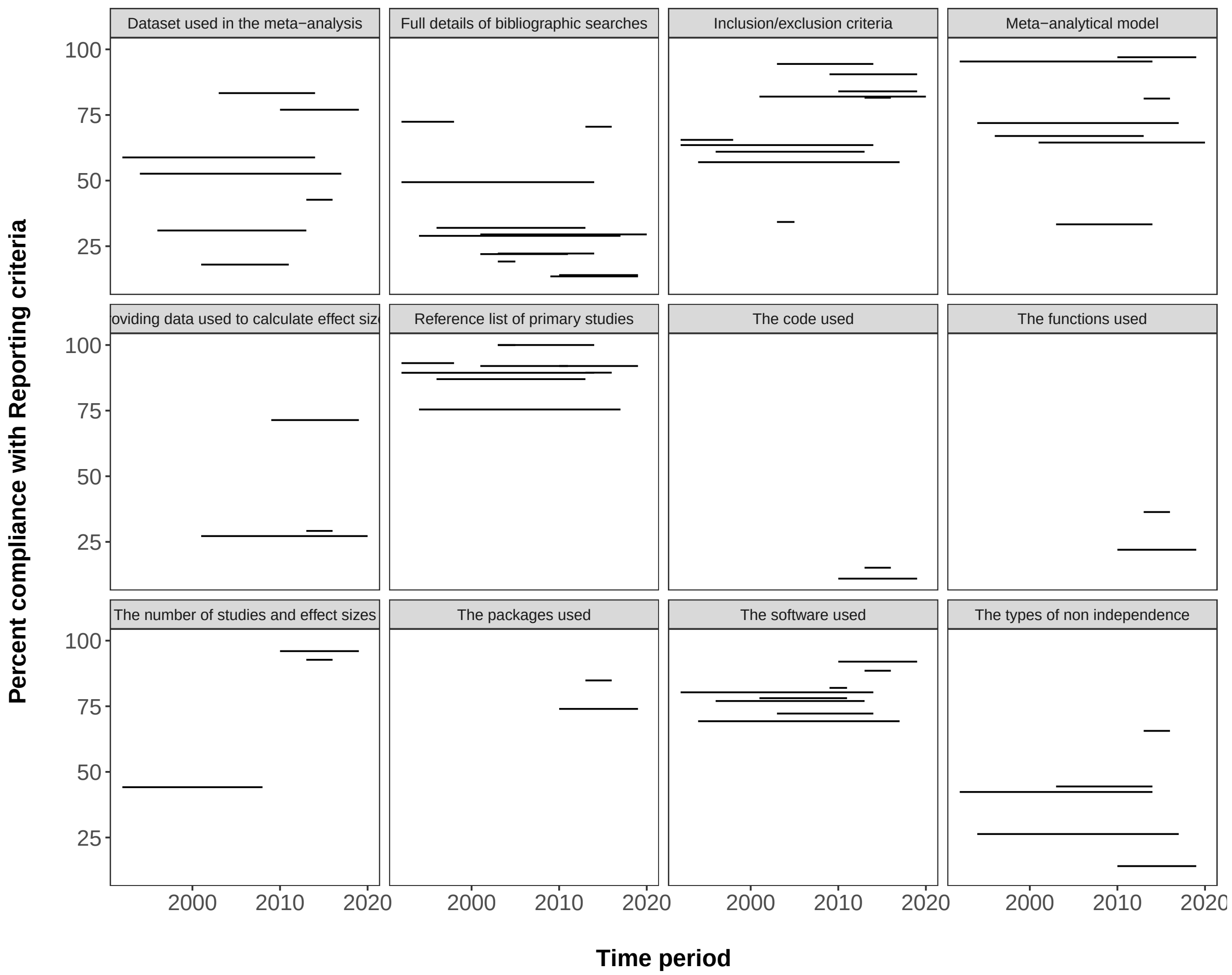

Fig S5. Percent of papers complying with Reporting criteria as a function of the time period analyzed by the review paper. Each panel represents a criterion. The line segment indicates the time period covered by each of the review papers that addressed a particular criterion.

```
# create Figure S6

fig_s6 <- ggplot(execution, aes(mid_point, percent)) +
  geom_segment(aes(x = first_year, y = percent,
                   xend = final_year, yend = percent), data = execution) +
  niceplot +
  facet_wrap(~ criteria) +
  #ggtitle("Execution") +
  labs(x= "Time period", y= "Percent compliance with Execution criteria") +
  niceplot

# save figure S6

ggsave("figures/Fig_S6.pdf", fig_s6, width = 10, height = 8, dpi = 300)
```

```
# display figure S6

fig_s6
```

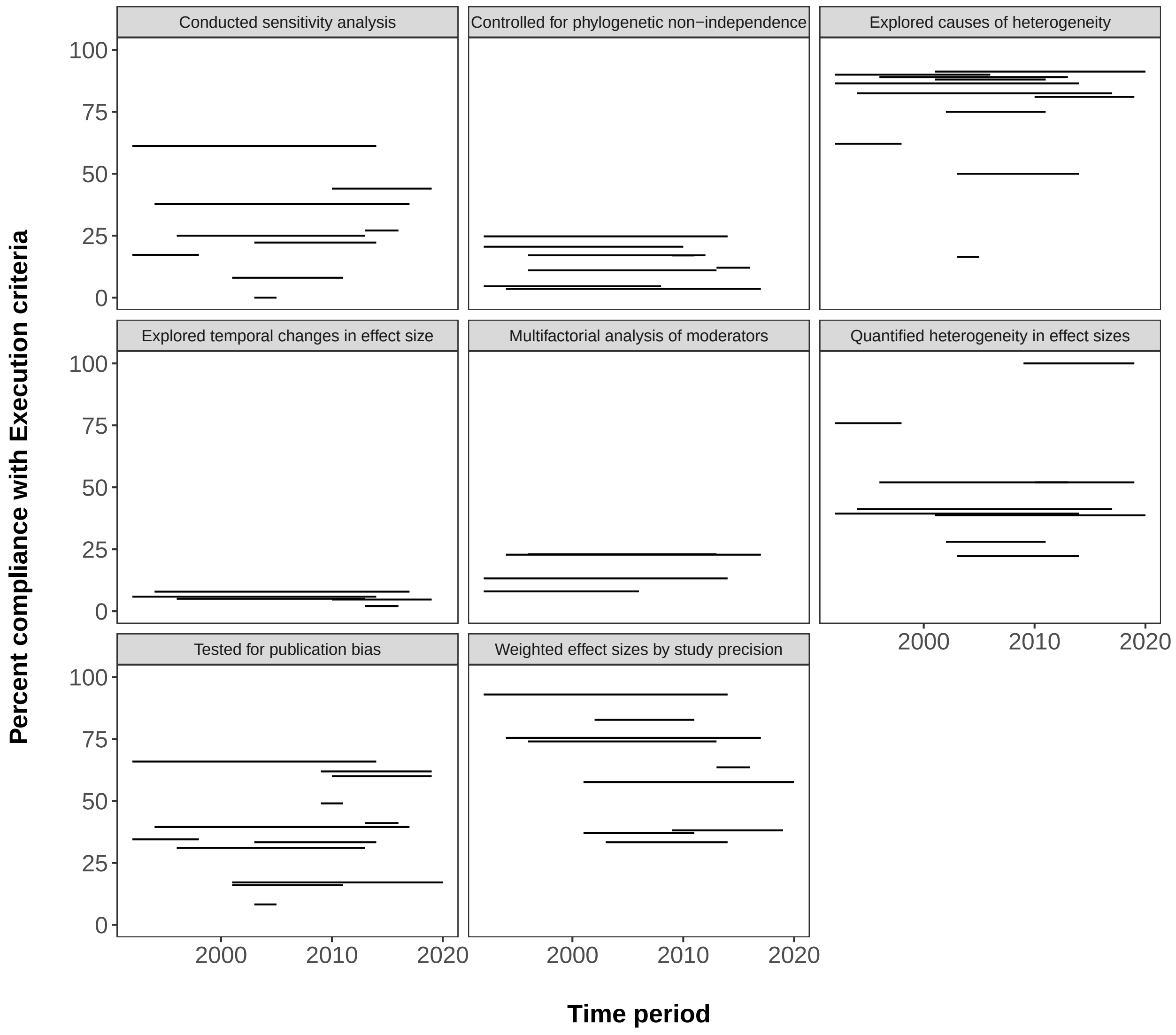

Fig S6. Percent of papers complying with Execution criteria as a function of the time period analyzed by the review paper. Each panel represents a criterion. The line segment indicates the time period covered by each of the review papers that addressed a particular criterion.
