# Supplementary material for: A meta-evaluation of the quality of reporting and execution in ecological meta-analyses
Source: PLoS One. 2023 Oct 12;18(10):e0292606. doi: 10.1371/journal.pone.0292606 (PMC10569516; doi:10.1371/journal.pone.0292606)
Supplement: S5 Appendix — Number of meta-analyses per journal that had been included in the meta-analysis reviews. Because the distribution is strongly right skewed (with most journals publishing a few meta-analyses), we display only the journals with at least 5 meta-analyses. (PDF) [file pone.0292606.s012.pdf]

Appendix S5

Paula Pappalardo, Chao Song, Bruce A. Hungate, Craig W. Osenberg

From: A meta-evaluation of the quality of reporting and execution in ecological meta-analyses

Setup

```r
knitr::opts_chunk$set(echo = T, eval = T, warning = F,
                      message = F, comment = "")

# load libraries we need

library(kableExtra)
library(tidyverse)
library(ggcharts)
library(rcartocolor)
library(readxl)
library(flextable)
```

Journals that most often publish meta-analyses

By pooling the reference list from all the review papers that made the references available (and without counting references twice), we were able to assess which journals tend to publish more meta-analyses. The top five journals were: 1) Ecology Letters (n= 91), 2) Global Change Biology (n= 89), 3) Ecology (n= 75), 4) Oecologia (n = 48), and American Naturalist (n = 43). Below you can see the number of meta-analyses per journal that had been included in the meta-analysis reviews. Because the distribution is strongly right skewed (with most journals publishing a few meta-analyses), we display only the journals with at least 5 meta-analyses.

```r
# function to select columns of interest

selectColumns <- function(mydf){
  mydf_ed <- mydf %>% select(first_author, year, journal)
  return(mydf_ed)
}

# function to add a standarized reference ID to compare accross reviews
addID <- function(mydf){
  mydf_ed <- mydf %>%
    mutate(ref_id = paste(str_to_upper(first_author), year,
                          str_to_upper(journal), sep = "_"),
           journal = str_to_upper(journal))
```

```r
  return(mydf_ed)
}


# load short journals dictionary

journal_dic <- read.csv("data/DataFiles_journal-names-dictionary.csv", as.is = T)
```

```r
# load references for each publication

arch <- as.data.frame(read_excel("data/DataFiles_list-of-references-in-Reviews.xlsx",
                                 sheet= "arch2015", range= cell_cols("A:D"))) %>%
  selectColumns() %>%
  addID()

cado <- as.data.frame(read_excel("data/DataFiles_list-of-references-in-Reviews.xlsx",
                                 sheet= "cado2012", range= cell_cols("A:D"))) %>%
  selectColumns() %>%
  addID()

cham <- as.data.frame(read_excel("data/DataFiles_list-of-references-in-Reviews.xlsx",
                                 sheet= "cham2012", range= cell_cols("A:C"))) %>%
  addID()

gate <- as.data.frame(read_excel("data/DataFiles_list-of-references-in-Reviews.xlsx",
                                 sheet= "gate2002", range= cell_cols("A:C"))) %>%
  addID()

jenn <- as.data.frame(read_excel("data/DataFiles_list-of-references-in-Reviews.xlsx",
                                 sheet= "jenn2012", range= cell_cols("A:B"))) %>%
  left_join(journal_dic, by = "journal_short") %>%
  rowwise() %>%
  mutate(year = as.numeric(str_extract(reference, "\\d{4}")[[1]]),
         first_author = str_split_fixed(reference, " ", 2)[,1]) %>%
  ungroup() %>%
  selectColumns() %>% addID()

kori <- as.data.frame(read_excel("data/DataFiles_list-of-references-in-Reviews.xlsx",
                                 sheet= "kori2012", range= cell_cols("A:C"))) %>%
  addID()

odea <- as.data.frame(read_excel("data/DataFiles_list-of-references-in-Reviews.xlsx",
                                 sheet= "odea2021", range= cell_cols("A:C"))) %>%
  addID()

papp <- as.data.frame(read_excel("data/DataFiles_list-of-references-in-Reviews.xlsx",
                                 sheet= "papp2020", range= cell_cols("A:C"))) %>%
  addID()

phil<- as.data.frame(read_excel("data/DataFiles_list-of-references-in-Reviews.xlsx",
                                sheet= "phil2012", range= cell_cols("A:C"))) %>%
  left_join(journal_dic, by = "journal_short") %>%
  selectColumns() %>% addID()
```

```r
robe <- as.data.frame(read_excel("data/DataFiles_list-of-references-in-Reviews.xlsx",
                                 sheet= "robe2006", range= cell_cols("A:C"))) %>%
  addID()

seni <- as.data.frame(read_excel("data/DataFiles_list-of-references-in-Reviews.xlsx",
                                 sheet= "seni2016", range= cell_cols("A:C"))) %>%
  addID()

vett <- as.data.frame(read_excel("data/DataFiles_list-of-references-in-Reviews.xlsx",
                                 sheet= "vett2013", range= cell_cols("A:C"))) %>%
  addID()

lodi_fe <- as.data.frame(read_excel("data/DataFiles_list-of-references-in-Reviews.xlsx",
                                    sheet= "lodi2021_fe", range= cell_cols("A:C"))) %>%
  mutate(first_author =  str_split_fixed(Citation, ",", 2)[,1],
         journal = ifelse(journal == "The American Naturalist",
                          "American Naturalist", journal)) %>%
  addID()

lodi_ee <- as.data.frame(read_excel("data/DataFiles_list-of-references-in-Reviews.xlsx",
                                    sheet= "lodi2021_ee", range= cell_cols("A:C"))) %>%
  addID()

beil <- as.data.frame(read_excel("data/DataFiles_list-of-references-in-Reviews.xlsx",
                                 sheet= "beil2022", range= cell_cols("A:D"))) %>%
  addID()

roma_a <- as.data.frame(read_excel("data/DataFiles_list-of-references-in-Reviews.xlsx",
                  sheet= "roma2021a", range= cell_cols("A:D"))) %>%
  addID()

roma_b <- as.data.frame(read_excel("data/DataFiles_list-of-references-in-Reviews.xlsx",
                  sheet= "roma2021b", range= cell_cols("A:C"))) %>%
  rowwise() %>%
  mutate(year = as.numeric(str_extract(Citation, "\\d{4}")[[1]])) %>%
  ungroup() %>%
  addID()
```

```r
# combine all unique ids (to avoid counting papers twice)

allids <- sort(unique(c(arch$ref_id, cado$ref_id, cham$ref_id, gate$ref_id,
              jenn$ref_id, kori$ref_id, odea$ref_id, papp$ref_id,
              phil$ref_id, robe$ref_id, seni$ref_id, vett$ref_id,
              lodi_fe$ref_id, lodi_ee$ref_id, beil$ref_id, roma_a$ref_id)))

# Compile all papers and create journal counts

alljournals <- data.frame(ids = allids) %>%
  mutate(journal = str_split_fixed(ids, "_", 3)[,3]) %>%
  add_count(journal) %>%
  distinct(journal, n) %>%
  arrange(desc(n)) %>%
  filter(n > 4)
```

```r
flextable(alljournals, cwidth = 3)
```

journal	n
ECOLOGY LETTERS	91
GLOBAL CHANGE BIOLOGY	89
ECOLOGY	75
OECOLOGIA	48
AMERICAN NATURALIST	43
OIKOS	41
CONSERVATION BIOLOGY	40
JOURNAL OF ECOLOGY	34
BIOLOGICAL CONSERVATION	34
AGRICULTURE, ECOSYSTEMS & ENVIRONMENT	33
NEW PHYTOLOGIST	28
PROCEEDINGS OF THE ROYAL SOCIETY B	27
JOURNAL OF APPLIED ECOLOGY	26
GLOBAL ECOLOGY AND BIOGEOGRAPHY	24
ECOLOGICAL APPLICATIONS	22
FOREST ECOLOGY AND MANAGEMENT	22
EVOLUTION	21
MOLECULAR ECOLOGY	21
BEHAVIORAL ECOLOGY	20
ANIMAL BEHAVIOUR	18
PLANT AND SOIL	17
ANNUAL REVIEW OF ECOLOGY, EVOLUTION, AND SYSTEMATICS	16
MARINE ECOLOGY PROGRESS SERIES	15
JOURNAL OF ANIMAL ECOLOGY	15
SOIL BIOLOGY & BIOCHEMISTRY	15
FUNCTIONAL ECOLOGY	14
TRENDS IN ECOLOGY & EVOLUTION	13
BIOLOGY LETTERS	11

journal	n
PROCEEDINGS OF THE NATIONAL ACADEMY OF SCIENCES USA	11
PLOS ONE	11
LAND DEGRADATION & DEVELOPMENT	10
JOURNAL OF EVOLUTIONARY BIOLOGY	10
BIOGEOSCIENCES	9
ECOLOGICAL MONOGRAPHS	9
AMERICAN JOURNAL OF BOTANY	9
ECOLOGY AND EVOLUTION	9
SCIENTIFIC REPORTS	8
ENVIRONMENTAL POLLUTION	8
SCIENCE	8
SCIENCE OF THE TOTAL ENVIRONMENT	8
BIOLOGICAL REVIEWS	8
ECOSYSTEMS	8
FIELD CROPS RESEARCH	8
FRESHWATER BIOLOGY	7
SOIL & TILLAGE RESEARCH	7
BEHAVIORAL ECOLOGY AND SOCIOBIOLOGY	7
CANADIAN JOURNAL OF FISHERIES AND AQUATIC SCIENCES	7
EVOLUTIONARY ECOLOGY	6
GLOBAL CHANGE BIOLOGY BIOENERGY	6
NATURE	6
BIOLOGICAL INVASIONS	6
ECOGRAPHY	6
PLANT, CELL AND ENVIRONMENT	5
RESTORATION ECOLOGY	5
PHILOSOPHICAL TRANSACTIONS OF THE ROYAL SOCIETY B	5
EVOLUTIONARY APPLICATIONS	5
ECOLOGICAL INDICATORS	5
PLANT	5

journal	n
CANADIAN JOURNAL OF FOREST RESEARCH	5
BIOGEOCHEMISTRY	5
