# Supplementary material for: A meta-evaluation of the quality of reporting and execution in ecological meta-analyses
Source: PLoS One. 2023 Oct 12;18(10):e0292606. doi: 10.1371/journal.pone.0292606 (PMC10569516; doi:10.1371/journal.pone.0292606)
Supplement: Attachment — Submitted filename: PONE-D-22-24320_Reviewer Comments.pdf [file pone.0292606.s019.pdf]

PLOS ONE

A meta-evaluation of the quality of reporting and execution in ecological meta-analyses

--Manuscript Draft--

**Manuscript Number:**	PONE-D-22-24320
**Article Type:**	Research Article
**Full Title:**	A meta-evaluation of the quality of reporting and execution in ecological meta-analyses
**Short Title:**	Quality of reporting and execution in ecological meta-analyses
**Corresponding Author:**	Paula Pappalardo, Ph.D<br>Smithsonian National Museum of Natural History<br>UNITED STATES
**Keywords:**	research synthesis; meta-analysis; non-independence; reproducibility; publication bias; sensitivity analysis; effect size; weighting
**Abstract:**	Quantitatively summarizing results from a collection of primary studies with meta-analysis can help answer ecological questions and identify knowledge gaps. The accuracy of the answers depends on the quality of the meta-analysis. We reviewed the literature assessing the quality of ecological meta-analyses to evaluate current practices and highlight the areas that need improvements. From each of 18 review papers that evaluated the quality of meta-analyses, we calculated the percentage of meta-analyses that met criteria related to specific steps taken in the meta-analysis process (i.e., execution) and the clarity with which those steps were articulated (i.e., reporting). We also re-evaluated all the meta-analyses available from Pappalardo et al. (2020) to extract new information on ten additional criteria and additional information on the recognition and treatment of non-independence. In general, we observed better performance for criteria related to reporting than for criteria related to execution; however, there was wide variation among criteria and meta-analyses. Low to moderate compliance with execution criteria was particularly striking for controlling for phylogenetic non-independence, exploring temporal trends in effect sizes, and conducting a multifactorial analysis of moderators. The additional analysis of non-independence showed that even though most meta-analyses included multiple effect sizes per study, only 66% acknowledged some type of non-independence. The types of non-independence reported were most often related to the design of the original experiment than related to other sources. We suggest that providing specific training and encouraging authors to follow the newly developed PRISMA EcoEvo checklist (O'Dea et al., 2021) can improve the quality of ecological meta-analysis.
**Order of Authors:**	Paula Pappalardo, Ph.D
	Chao Song
	Bruce A. Hungate
	Craig W. Osenberg
**Additional Information:**	
**Question**	**Response**
**Financial Disclosure**<br><br>Enter a financial disclosure statement that describes the sources of funding for the work included in this submission. Review the submission guidelines for detailed requirements. View published research articles from *PLOS ONE* for specific examples.<br><br>This statement is required for submission	CWO was supported through grant DE-SC-0010632 from the U.S. Department of Energy (https://www.energy.gov/) and grant DEB-1655426 from the National Science Foundation (https://www.nsf.gov/). The funders had no role in study design, data collection and analysis, decision to publish, or preparation of the manuscript.

Powered by Editorial Manager® and ProduXion Manager® from Aries Systems Corporation

and **will appear in the published article** if the submission is accepted. Please make sure it is accurate.<br><br>**Unfunded studies**<br>Enter: *The author(s) received no specific funding for this work.*<br><br>**Funded studies**<br>Enter a statement with the following details:<br>• Initials of the authors who received each award<br>• Grant numbers awarded to each author<br>• The full name of each funder<br>• URL of each funder website<br>• Did the sponsors or funders play any role in the study design, data collection and analysis, decision to publish, or preparation of the manuscript?<br>• **NO** - Include this sentence at the end of your statement: *The funders had no role in study design, data collection and analysis, decision to publish, or preparation of the manuscript.*<br>• **YES** - Specify the role(s) played.<br><br>* typeset	
**Competing Interests**<br><br>Use the instructions below to enter a competing interest statement for this submission. On behalf of all authors, disclose any competing interests that could be perceived to bias this work—acknowledging all financial support and any other relevant financial or non-financial competing interests.<br><br>This statement is required for submission and **will appear in the published article** if the submission is accepted. Please make sure it is accurate and that any funding sources listed in your Funding Information later in the submission form are also declared in your Financial Disclosure statement.<br><br>View published research articles from *PLOS ONE* for specific examples.	The authors have declared that no competing interests exist.

Powered by Editorial Manager® and ProduXion Manager® from Aries Systems Corporation

**NO authors have competing interests**<br><br>Enter: *The authors have declared that no competing interests exist.*<br><br>**Authors with competing interests**<br><br>Enter competing interest details beginning with this statement:<br><br>*I have read the journal's policy and the authors of this manuscript have the following competing interests: [insert competing interests here]*<br><br>* typeset	
**Ethics Statement**<br><br>Enter an ethics statement for this submission. This statement is required if the study involved:<br><br>• Human participants<br>• Human specimens or tissue<br>• Vertebrate animals or cephalopods<br>• Vertebrate embryos or tissues<br>• Field research<br><br>Write "N/A" if the submission does not require an ethics statement.<br><br>General guidance is provided below. Consult the submission guidelines for detailed instructions. **Make sure that all information entered here is included in the Methods section of the manuscript.**	N/A

Powered by Editorial Manager® and ProduXion Manager® from Aries Systems Corporation

**Format for specific study types**<br><br>**Human Subject Research (involving human participants and/or tissue)**<br>• Give the name of the institutional review board or ethics committee that approved the study<br>• Include the approval number and/or a statement indicating approval of this research<br>• Indicate the form of consent obtained (written/oral) or the reason that consent was not obtained (e.g. the data were analyzed anonymously)<br><br>**Animal Research (involving vertebrate animals, embryos or tissues)**<br>• Provide the name of the Institutional Animal Care and Use Committee (IACUC) or other relevant ethics board that reviewed the study protocol, and indicate whether they approved this research or granted a formal waiver of ethical approval<br>• Include an approval number if one was obtained<br>• If the study involved *non-human primates*, add *additional details* about animal welfare and steps taken to ameliorate suffering<br>• If anesthesia, euthanasia, or any kind of animal sacrifice is part of the study, include briefly which substances and/or methods were applied<br><br>**Field Research**<br><br>Include the following details if this study involves the collection of plant, animal, or other materials from a natural setting:<br>• Field permit number<br>• Name of the institution or relevant body that granted permission	
**Data Availability**<br><br>Authors are required to make all data underlying the findings described fully available, without restriction, and from the time of publication. PLOS allows rare exceptions to address legal and ethical concerns. See the PLOS Data Policy and FAQ for detailed information.	Yes - all data are fully available without restriction

Powered by Editorial Manager® and ProduXion Manager® from Aries Systems Corporation

A Data Availability Statement describing where the data can be found is required at submission. Your answers to this question constitute the Data Availability Statement and **will be published in the article**, if accepted.<br><br>**Important:** Stating 'data available on request from the author' is not sufficient. If your data are only available upon request, select 'No' for the first question and explain your exceptional situation in the text box.<br><br>Do the authors confirm that all data underlying the findings described in their manuscript are fully available without restriction?	
**Describe where the data may be found in full sentences. If you are copying our sample text, replace any instances of XXX with the appropriate details.**<br><br>• If the data are **held or will be held in a public repository**, include URLs, accession numbers or DOIs. If this information will only be available after acceptance, indicate this by ticking the box below. For example: *All XXX files are available from the XXX database (accession number(s) XXX, XXX.)*.<br>• If the data are all contained **within the manuscript and/or Supporting Information files**, enter the following: *All relevant data are within the manuscript and its Supporting Information files.*<br>• If neither of these applies but you are able to provide **details of access elsewhere**, with or without limitations, please do so. For example:<br><br>*Data cannot be shared publicly because of [XXX]. Data are available from the XXX Institutional Data Access / Ethics Committee (contact via XXX) for researchers who meet the criteria for access to confidential data.*<br><br>*The data underlying the results presented in the study are available from (include the name of the third party*	All relevant data are within the manuscript and its Supporting Information files.

and contact information or URL).<br>• This text is appropriate if the data are owned by a third party and authors do not have permission to share the data.<br><br>* typeset	
Additional data availability information:	

Powered by Editorial Manager® and ProduXion Manager® from Aries Systems Corporation

Manuscript
Click here to access/download;Manuscript;Quality of reporting in meta-analysis_MS.docx

A meta-evaluation of the quality of reporting and execution in ecological meta-analyses

Paula Pappalardo[1*], Chao Song[2], Bruce A. Hungate[3], Craig W. Osenberg[4]

1. Department of Invertebrate Zoology, Smithsonian National Museum of Natural History, Washington, DC, USA

2. State Key Laboratory of Grassland Agro-ecosystems and College of Ecology, Lanzhou University, Lanzhou, Gansu 730000, China

3. Department of Biological Sciences, Northern Arizona University, Flagstaff, Arizona

4. Odum School of Ecology, University of Georgia, Athens, GA 30602, USA

* Corresponding author

Abstract

Quantitatively summarizing results from a collection of primary studies with meta-analysis can help answer ecological questions and identify knowledge gaps. The accuracy of the answers depends on the quality of the meta-analysis. We reviewed the literature assessing the quality of ecological meta-analyses to evaluate current practices and highlight the areas that need improvements. From each of 18 review papers that evaluated the quality of meta-analyses, we calculated the percentage of meta-analyses that met criteria related to specific steps taken in the meta-analysis process (i.e., execution) and the clarity with which those steps were articulated (i.e., reporting). We also re-evaluated all the meta-analyses available from Pappalardo et al. (2020) to extract new information on ten additional criteria and additional information on the recognition and treatment of non-independence. In general, we observed better performance for criteria related to reporting than for criteria related to execution; however, there was wide variation among criteria and meta-analyses. Low to moderate compliance with execution criteria was particularly striking for controlling for phylogenetic non-independence, exploring temporal trends in effect sizes, and conducting a multifactorial analysis of moderators. The additional analysis of non-independence showed that even though most meta-analyses included multiple effect sizes per study, only 66% acknowledged some type of non-independence. The types of non-independence reported were most often related to the design of the original experiment than related to other sources. We suggest that providing specific training and encouraging authors to follow the newly developed PRISMA EcoEvo checklist (O'Dea et al., 2021) can improve the quality of ecological meta-analysis.

Introduction

Meta-analyses evaluate summary statistics from primary studies to obtain aggregate effects, assess the heterogeneity of those effects, and ascertain possible causes of the observed heterogeneity. For example, meta-analysis has been used to quantify the strength of density-dependence [1], to assess the response of ecosystems to climate change [2], and to evaluate the performance of different management strategies [3]. Through synthesis, meta-analysis not only advances basic ecological theory, but also facilitates the application of ecological data to inform environmental policy [4]. Moreover, meta-analysis can help identify knowledge gaps, and thus direct new research endeavors [5]. Along with these benefits, the number of published meta-analyses is rapidly increasing [6,7], due to increased data availability, and pressing ecological questions that require synthetic research.

Despite their importance and wide application, meta-analyses vary in the quality of execution, and the subsequent reporting of meta-analytic methods is highly variable [5,8–10]. If the quality of meta-analyses is poor, it is hard to know if “biological meta-analysis embodies ‘mega-enlightenment’, a ‘mega-mistake’, or something in between” [11]. One of the main issues that can prevent readers from evaluating the overall quality of a published meta-analysis is the lack of details describing each step in the meta-analysis (usually referred to as reporting quality). Poor reporting quality hinders the readers from assessing if the meta-analysis was executed properly and the results are reliable. New methodological guidelines specifically designed for ecology and evolutionary biology [PRISMA-EcoEvo, 12] provide authors, reviewers, and editors with a checklist of items with the goal of improving the overall quality of ecological meta-analysis. Wide adoption of these guidelines could greatly improve the quality of meta-analyses in ecology and

evolutionary biology. Accessing the current status of compliance with the recommended best practice in ecological meta-analyses and identifying places that need improvements is thus a necessary first step to guide the meta-analytic community towards more robust inference.

In this paper, we reviewed the literature assessing the quality of ecological meta-analysis and collected new data to evaluate current practices and highlight the areas that need more work. First, we compiled information from 18 studies in the last 20 years (between 2002 and 2022) that reviewed the quality of meta-analyses in ecology, evolution and related fields. These papers provide different insights on the compliance with different standards of reporting quality and with recommended steps that should be part of a meta-analysis. Second, we evaluated the recognition and treatment of non-independence for the ecological meta-analyses included in Pappalardo et al. [10], and extracted new data on quality criteria to compare it with the other reviews. Finally, we summarized the level of compliance for different quality criteria across the synthetic reviews and the new data.

Methods

To evaluate current practices when conducting and reporting ecological meta-analyses, we surveyed the literature for quantitative assessments on criteria previously identified as best practices in meta-analysis. These criteria fall into two broad categories: 1) execution (i.e., methodological issues), and 2) reporting. Both categories of criteria aim to ensure appropriate and reproducible results. Our list of criteria was informed by Koricheva & Gurevitch [5, Table 3] and the PRISMA EcoEvo checklist [12]. To find relevant papers, we first performed a exploratory search in Google Scholar, using combinations of keywords including “meta-analysis”, “review”,

“quality”, “ecology”, “evolution”. We then searched the Core Collection of the ISI Web of Science database including articles and reviews within the “Ecology”, “Evolutionary Biology”, “Biodiversity Conservation” and “Plant Sciences” categories (last search update on Sep 16, 2022). We used a search string for TOPIC as: ([“meta-analyses” OR “metaanalyses” OR “meta analyses"] AND [“quality” OR “performance criteria”] AND ["reporting"]). The search resulted in 751 citations. We supplemented those with 11 articles published in the ‘‘Meta-analytic insights into evolutionary ecology’’ Special Issue of Evolutionary Ecology (2012, Volume 26, Issue 5), and 3 articles found by being referenced by other articles or presented in scientific talks; the total number of papers screened (using the *metagear* [13] R package) was 765. A PRISMA plot made with the R package *PRISMAstatement* [14] is presented in Fig 1 and details the number of papers in each screening step.

Fig 1. PRISMA diagram.
The PRISMA diagram details the relevant literature sources identified, screened, excluded, assessed, and selected for the final analysis. Of the 19 final papers we compiled information from, we noticed that two of them review quality from a similar pool of papers (Romanelli et al., [25], Romanelli et al.,[26]). Because of this, we excluded Romanelli et al. [26] from the final comparison.

We compiled information from 19 papers (Table 1) that passed the inclusion criteria of having quantitative data on the quality of reporting or execution of meta-analysis in any of the criteria previously identified as target. A list of criteria and which paper contributed to each of them is provided in Table 2. We noticed that two of the reviews [25, 26] compiled information from a similar pool of studies in restoration ecology, so we only extracted information for 18 papers, excluding Romanelli et al. [26] from the final comparison. Because different review papers used slightly different criteria in their reviews, we matched similar criteria; details on which information was extracted for each paper and criteria is provided in Appendix A. We obtained the

proportion of the meta-analyses that comply with a particular criterion (data available in supplementary data files "compilation-of-previous-review-papers"). For the final analysis, we included only those criteria for which we could gather information from at least two review papers.

Table 1. Compilation of papers that reviewed the quality of reporting in ecological meta-analyses. The "Review ID" (first author initials plus publication year) was used to identify review papers in Fig 1, Fig 2, and tables and figures in the Supplementary Material. Lodi et al., 2021 reviewed the quality of meta-analysis in two topic areas, we identified them with an additional code (lodi2021_fe and lodi2021_ee). There are two Romanelli et al., 2021 publications, we separated them as roma2021a and roma2021b, and extracted data from roma2021a because it had information for more of our target quality criteria and both publications reviewed the same pool of meta-analyses.

Publication	Review ID	Area	Time period	Number of meta-analysis papers reviewed
Archmiller et al., 2015 [15]	arch2015	molecular ecology	2003-2014	18
Beillouin et al., 2022 [16]	beil2022	biodiversity conservation, ecology; environmental sciences	2001-2020	217[a]
Cadotte et al., 2012 [6]	cado2012	ecology	1992-2008	240
Chamberlain et al., 2012 [17]	cham2012	ecology and evolutionary biology	1992-2010	56
Chaudhary et al., 2010 [18]	chau2010	ecology	1992-2006	188
Gates, 2002 [8]	gate2002	ecology	1992-1998	29
Jennions et al., 2012 [19]	jenn2012	sexual selection	1996-2012	94

Koricheva & Gurevitch, 2014 [5]	kori2014	plant ecology	1996-2013	322
Lodi et al., 2021 [20]	lodi2021_fe	freshwater ecology	1994-2017	114
	lodi2021_ee	ecology and evolution	1992-2014	86[b]
Nakagawa & Santos, 2012 [21]	naka2012	ecology and evolution	2009-2011	100
Nakagawa et al., 2022[c] [22]	naka2022	ecology and evolutionary biology	2010-2019	102
ODea et al., 2021 [12]	odea2021	ecology and evolutionary biology	2010-2019	102
Pappalardo et al., 2020 [10]	papp2020	ecology, climate change	2013-2016	96
Philibert et al., 2012 [23]	phil2012	agronomy	2001-2011	73[d]
Roberts et al., 2006 [24]	robe2006	conservation, ecology, and environmental management	2003-2005	73
Romanelli et al., 2021 [25]	roma2021a	restoration ecology	2009-2019	63
Romanelli et al., 2021 [26]	roma2021b	restoration ecology	2009-2019	52
Senior et al., 2016 [27]	seni2016	ecology and evolution	1992-2014	325

Vetter et al., 2013 [28]	vett2013	ecology and conservation biology	2002-2011	133 and 83[e]

[a] For Beillouin et al., (2022), we counted the number of meta-analysis provided in the supplementary data file in the “retained meta-analyses” Table, even though we noticed the main text mentioned 196 meta-analyses. Most of the percentages mentioned in the main text agreed with the total being 217 based on the quality rating presented in the supplementary data file.

[b] Lodi et al., (2021) reviewed n = 86 papers from the n= 325 papers in Senior et al., 2016 for their subset of meta-analysis in ecology and evolution (they used the papers that Senior et al., 2016 included in their second order meta-analysis). We only collected quality data from Lodi et al., 2021 that was not already addressed by Senior et al., (2016).

[c] Nakagawa et al., (2022) reviewed the compilation of papers by O’Dea et al., (2021) extracting additional information for our criteria of interest.

[d] Philibert et al. (2012) analyzed 73 meta-analyses from 55 papers.

[e] Vetter et al. (2013) reviewed a first round 133 papers, from which they quantified the percentage that did not report using weights. Then, from the 83 papers that passed their filter to be a “sound” meta-analysis, they quantified the percentage of papers that quantified and explored heterogeneity.

If there is overlap in the meta-analyses reviewed by these papers, comparisons between review papers may not be independent; however, because each review used a different set of search algorithms and often targeted a specific topic, such overlap is likely small. We quantified the overlap between meta-analysis reviews for all cases in which the full list of references was available (in the main text or in the supplementary material), or when the authors replied to our requests for this information. We used the first author’s last name, journal, and year as the identification string to measure overlap in the number of publications shared between review papers.

In addition, because we had access to the full set of meta-analyses reviewed by Pappalardo et al. [10], we expanded on their results by adding additional criteria evaluated here (Table 2). Pappalardo et al. [10] analyzed 96 meta-analyses related with global change (PRISMA diagram

available in their Fig S1). For the criteria related to Reporting, we collected new information on: inclusion/exclusion criteria, the number of papers and the number of effect size estimates, the types of non-independence, identifying the statistical package used, identifying the specific functions used for the analyses, and providing the code that was used (when applicable). For the criteria related to Execution, we evaluated if the publication explored temporal changes in effects, conducted sensitivity analyses, controlled for phylogenetic non-independence, and tested for publication bias. More details on the calculations for each criterion are provided in Appendix A. When a publication acknowledged non-independence (e.g., described some type of non–independence), we also recorded the source of the non-independence being acknowledged, if the authors attempted to account for it, and the methods used to address non-independence. The source of non-independence coded as "sample" refers to non-independence among observed effect sizes arising from non-independent within-study error (e.g., multiple measurements of the same individual, shared control or treatments); and were coded as "study" when the non-independence arises from study-level correlation (e.g., multiple effect sizes from each publication which could generate random paper effects).

To code if a publication addressed non-independence, we used: "yes", when the publication described one or more sources of non-independence and addressed at least one; "partially" when the publication mentioned more than one source of independence, but did not address all of them; and "no" when the publication did not address non-independence. We coded the methods used to deal with non-independence as: 1) "average", when the non-independent values were averaged [e.g., 29: averaged repeated measurements across study durations,30: averaged across species]; 2) "choose", when the authors chose one value from multiple non-independent values [e.g., 31: used last sampling point,32: used one response variable per study]; and 3) "model", when the authors

accounted for non-independence within the meta-analytic model [e.g., 33: included paper ID as a random effect,34: included variance-covariance matrix obtained from phylogenetic distances]; and 4) “tested”, when non-independence was assessed, found not to be demonstrable, and was subsequently ignored [e.g., 35–37]. If a test was done and non-independence was supported, then the paper was coded according to the method used to address non-independence, and not as “tested” [e.g., 38].

We compiled the percentage of compliance for each criterion from the review papers and the new data, separating the criteria into Reporting and Execution types. We classified performance for each criterion as “high” when the percentage of papers complying with a criterion was ≥75%, “moderate” when compliance was ≥50% but <75%, “low” when compliance was ≥25% but <50%, and “very low” when compliance was <25%. The data collected is provided as supplementary data files. We analyzed and visualize data using the R software [39] and packages *scales* [40], *flextable* [41], *pander* [42], *kableExtra* [43], *readxl* [44], *ggcharts* [45], and *tidyverse* [46]. The code used to compile information, re-analyze data, and combine the final dataset used for analysis, is available in the supplementary material.

Table 2. List of Reporting and Execution criteria compiled from reviews of meta-analyses.

Publications are identified using the Review ID provided in Table 1, and marked “yes” if they provided information for a specific criterion. For Pappalardo et al. [10], we indicated “yes” when we reanalyzed their published dataset, and “added” when we re-reviewed the meta-analyses to compile new data. Blank entries represent cases where data was not available for a specific criterion (likely because it was not the focus of the authors’s review).

**Criteria**	papp2020	odea2021	kori2014	lodi2021_fe	lodi2021_ee	arch2015	phil2012	beil2022	gate2002	roma2021a	robe2006	seni2016	naka2012	vett2013	cado2012	chau2010	jenn2012	naka2022	cham2012
Reporting																			
Full details of bibliographic searches	yes	yes	yes	yes	yes	yes	yes	yes	yes	yes	yes								
Inclusion/exclusion criteria	*added*	yes	yes	yes	yes	yes		yes	yes	yes	yes								
Reference list of primary studies	yes	yes	yes	yes	yes	yes	yes		yes		yes								
Meta-analytical model	yes	yes	yes	yes		yes		yes				yes							
Dataset used in the meta-analysis	yes	yes	yes	yes	yes	yes	yes												
Data used to calculate effect sizes (raw data)	yes							yes		yes									
The number of papers and the number of effect size estimates	*added*	yes													yes				
The software used	yes	yes	yes	yes		yes	yes					yes	yes						
The packages used (if applicable)	*added*	yes																	
The functions used (if applicable)	*added*	yes																	
The code used (if applicable)	*added*	yes																	
The types of non-independence	*added*	yes		yes	yes	yes													
Execution																			
Weighted effect sizes by study precision	yes		yes	yes	yes	yes	yes	yes		yes				yes					
Quantified heterogeneity in effect		yes	yes	yes		yes	yes	yes	yes	yes	yes	yes		yes					

sizes																			
Explored causes of heterogeneity	yes	yes	yes	yes		yes		yes	yes			yes		yes		yes			
Conducted multifactorial analysis of moderators			yes	yes								yes				yes			
Tested for publication bias	*added*	yes	yes	yes	yes	yes	yes	yes	yes	yes	yes		yes						
Conducted sensitivity analysis	*added*	yes	yes	yes	yes	yes	yes		yes		yes								
Explored temporal changes in effect size	*added*		yes	yes	yes													yes	
Controlled for phylogenetic non-independence	*added*		yes	yes	yes								yes		yes		yes		yes

Results

Overlap between review papers

The overlap between review papers was in general low. For Reporting criteria, the median number of shared papers was 2 and the mean 3; for Execution criteria, the median number of shared papers was 2 and the mean 4.7. In the Supplementary Material (section “Overlap between review papers”) we included the overlap matrices for each quality criterion that show the number of papers that overlap between each review paper, and the distribution of number of papers shared (Fig S2, S3). The two instances with largest overlap were: 1) overlap of 77 papers between Koricheva & Gurevitch [5] and Senior et al., [27], representing a 23.9% overlap for the “Meta-analytical model”, “The software used”, “Quantified heterogeneity in effect sizes”, “Tested for publication bias”, and “Multifactorial analysis of moderators criteria” criteria; 2) overlap of 74 papers between Koricheva & Gurevitch [5] and Cadotte et al., 2012 [6], representing 30.8% overlap for the “Controlled for phylogenetic non-independence” criteria.

Compliance with Reporting and Execution criteria

In our compilation of synthesis reviews, we found wide variability in the compliance within and between the different quality criteria. We did not observe any clear differences among different subdisciplines (Figs. S4, S5), nor did we observe any temporal trends in compliance (Figs. S6, S7).

In general, we observed better compliance with criteria related to Reporting (Fig 2) than Execution (Fig 3). Across reviews, we observed high to moderate compliance with Reporting criteria such as: providing the list of references (Fig 2F), specifying the meta-analytic model (Fig

2D), detailing inclusion/exclusion criteria (Fig 2C), and identifying the packages (Fig 2J) and software (Fig 2K) used. On the other hand, Reporting criteria such as including full details on the literature search (Fig 2B), providing the data used to calculate effect sizes (Fig 2E), and providing the analytic code (Fig 2G) and functions (Fig 2H) used exhibited very low, to moderate compliance.

Fig 2: Percent of papers complying with Reporting criteria.

The percent of papers complying with each criterion is plotted for each synthesis paper. The colors indicate the overall performance for each criterion coded as: "high" (percentage compliance ≥ 75%), "moderate" (50 ≤ percentage compliance < 75), "low" (25 ≤ percentage compliance < 50), and "very low" (percent compliance < 25%). The Review ID corresponds to the papers listed in Table 1.

For the Execution criteria, there was lower compliance with criteria such as conducting sensitivity analysis (Fig 3A), controlling for phylogenetic non-independence (Fig 3B), exploring temporal changes in effect sizes (Fig 3D), conducting a multifactorial analysis of moderators (vs. multiple single factor analyses) (Fig 3E), and testing for publication bias (Fig 3G). In contrast, most papers explored the possible causes of heterogeneity (Fig 3C). For the Execution criteria of weighing effect sizes by study precision (Fig 3H) and quantifying heterogeneity in effect sizes (Fig 3F), compliance was highly variable.

Fig 3: Percent of papers complying with Execution criteria (i.e., recommendations during data analysis).

The percent of papers complying with each criterion is plotted for each synthesis paper. The colors indicate the overall performance for each criterion coded as: "high" (percentage compliance ≥ 75%), "moderate" (50 ≤ percentage compliance < 75), "low" (25 ≤ percentage compliance < 50), and "very low" (percent

compliance < 25%). The Review ID corresponds to the papers listed in Table 1. In panel (A), Roberts [24] evaluated sensitivity analysis, and found 0% of papers reporting it.

Non-independence

In our review of the meta-analyses compiled by Pappalardo et al., [10], we found that in all meta-analyses but one, the number of effect sizes was larger than the number of papers (Fig 4). This variation was often of several orders of magnitude (Fig 4, note the log scale in both axis). This suggests the possibility of non-independence as effect sizes derived from the same source paper are more likely to be similar than those coming from different papers. 66% of the meta-analysis acknowledged some type of non-independence (Fig 5A). The source of non-independence acknowledged most often (68% of the time) was related to the design of the original experiment (e.g., a common control used for different treatments) and how data was collected (Fig 5B). Acknowledging non-independence from other sources of correlation (e.g., multiple experiments per publication) was less common (36% of the time, Fig 5B). The majority of papers (95%) that did acknowledge some source of non-independence addressed it (Fig 5C). The most common ways that non-independence was addressed (Fig 5D) were: choose (50%), average (27%), model (14%) and tested (11%), with some papers using a combination of these approaches.

Fig 4: Relationship between the number of papers and the number of effect sizes included in ecological meta-analysis.

This plot was made by re-analyzing the compilation of meta-analyses by Pappalardo et al. [10]. Note that the axis are in log scale to accommodate two studies with extreme number of effect sizes. One is a study reporting 52 papers that did not specify the number of effect sizes and provided a dataset with 46,347 rows [47]. The other is a study that analyzed 1,785 papers and reported a total of 32,567 effects for one of their meta-analyses [48]. The dashed line indicates the abline for x = y.

Fig 5: Percent of papers that acknowledge non-independence, addressed it, and which methods they used to deal with non-independence.

(A) Acknowledged at least one type of independence in their data ("yes") or did not acknowledge non-independence ("no"). (B) For the papers that did acknowledge non-independence, these are the sources of non-independence classified as "study" or "sample". (C) The percent of papers that addressed all of the types of non-independence mentioned ("yes"), a subset of the factors mentioned ("partially"), or did not address non-independence ("no"). (D) For the papers that did address non-independence, we show the methods used to address non-independence, classified as: "choose", when the authors chose one value from multiple non-independent values; "average", average, when the non-independent values were averaged; "model", when the authors accounted for non-independence within the meta-analytic model, and "tested" when the authors tested for the effects of non-independence and decided they were not significant. Please note that the papers that used more than one method (or source) were counted in each category, so the percentages between levels of Fig B and D do not add exactly to 100%.

Discussion

Compliance with Reporting criteria

Even though there was overall good compliance for Reporting criteria (e.g., providing the list of primary papers included in the meta-analysis), many issues remain widespread. Meta-analysis papers were less consistent in their reporting of information that can critically affect the results of a meta-analysis [e.g., the inclusion/exclusion criteria 49]. Even minimal information such as the number of papers and effect sizes were not always included; for example, the review by Cadotte [6] showed that fewer than 50% of the meta-analyses reported this basic information. Many of these meta-analyses are not reproducible, because relatively few of the meta-analyses provided the data used to conduct their analyses (e.g., effect sizes, variances, moderators), or specified the model used to analyze the data (e.g., random-effects model). Similarly, for the meta-analysis that reported using a programming language, very few report the specific functions used for data analysis or provide their code, which are essential for reproducibility. Low code-sharing is not

exclusive to meta-analysis; even for research articles published in ecological journals that encourage or mandate code-sharing, only 27% provide all or some of the code used for the analysis [50]. Making the data available provides great benefits for the research community, because it can be useful for meta-research or integrative research that combines that data in some novel way [51]. To encourage code and data sharing, journals can develop incentives for authors to publish their datasets or code (e.g., a discount on open access fees for authors sharing data and code). Most data repositories will provide a separate DOI for the dataset that can be properly cited. Most importantly, as reporting practices improve and data become available, reproducibility will improve. Achieving at least computational reproducibility will ensure results are robust and credible. This is particularly important for researchers working in applied science and conservation [52].

Having common accepted guidelines for meta-analysis could be a useful tool to improve quality of meta-analysis although empirical research on this topic often gives mixed results. Even before the PRISMA guidelines were initially developed [in 2009 by 53], systematic reviews in the medical field showed higher reporting quality compared with literature reviews in ecological meta-analysis [8,24]. This was likely due to the early guidelines for systematic reviews in the medical field using a standard set of methods developed by the Cochrane Collaboration [54]. How much the PRISMA guidelines improved the quality of reporting in medical meta-analysis is not clear. Some papers report a moderate increase in reporting quality after the publication of PRISMA guidelines [55], others report no change [56, only reviewed abstracts]. For medical meta-analyses, the papers that compared self-reporting versus journals endorsing PRISMA found only higher quality of reporting when the journals endorse and implement the PRISMA guidelines [57,58]. In their synthesis review of meta-analyses in ecology and evolution O'Dea et al., [12] showed that

the meta-analysis that reported to have followed some guidelines tended to have higher quality ratings. Lodi et al., [20] found higher quality ratings on meta-analyses in freshwater ecology from more recent years and suggest that previous papers on reporting guidelines are the reason for the improvement. We think that the recently published PRIMA Eco-Evo guidelines [12] could generate a bigger impact in improving the quality of reporting if journals require those guidelines for submission of meta-analyses. Some journals such as PLOS ONE already have a structure in place to detect if certain key aspects of a self reporting meta-analysis are present (e.g., a PRISMA plot). By pooling the references from all the review papers we analyzed, we can highlight the five journals that publish the highest number of meta-analysis and that will benefit the most of implement the PRISMA Eco-Evo guidelines in their submission process: 1) Ecology Letters (n= 91), 2) Global Change Biology (n= 89), 3) Ecology (n= 75), 4) Oecologia (n = 48), and American Naturalist (n = 43). We list all the journals with at least 5 meta-analyses across reviews in Supplementary Table S1.

Compliance with Execution criteria

The overall low compliance with Execution criteria suggests that most meta-analyses do not follow recommended methods. Below we discuss some of the topics usually associated with the recommended steps for a meta-analysis.

Weighting. One of the advantages of meta-analysis is that effect sizes are conventionally weighted by the precision of the observed effect size. Our compilation of reviews showed that the percentage of papers that weighted the effect sizes varied widely (from 33% to 93%). By reanalyzing the meta-analyses from Pappalardo et al. [10], we found that only 42% weighted by the inverse of the variance, 6% weighted by sample size, 16% used some non-traditional weight,

and 36% of the meta-analyses did not weight effect sizes in any way to account for variation in their precision or quality. Several papers used unweighted analyses because of incomplete reporting in the primary publications (e.g., they did not report standard deviations or sample sizes), and they did not want to greatly reduce the number of studies by using a weighted meta-analysis. New imputation techniques to estimate standard deviations could be a good alternative that allows conducting a weighted meta-analysis without throwing away data [59]. However, although weighting is generally recommended for meta-analysis because it is expected to yield more precise estimates (and is one of the criteria in PRISMA-EcoEvo), unweighted analyses could provide results as reliable as those obtained using weighted analyses, particularly when among-study variance is large relative to within-study variances (Song et al, pers. comm.). Thus, weighting is not necessarily a requirement of a well-executed meta-analysis. Conducting a sensitivity analysis with a smaller dataset that allows to compare unweighted and weighted meta-analyses can be a way to check if results are consistent [60].

Heterogeneity. A central purpose of ecological meta-analysis is to quantify heterogeneity and explore its causes. A fixed-effects model assumes no heterogeneity among effect sizes, has been discouraged for ecological meta-analysis [61] and its use seems to be declining [27]. In fact, we showed (for most reviews) high compliance in exploring the causes of heterogeneity using explanatory variables (i.e., moderators), sometimes evaluated graphically. However, even when heterogeneity is addressed using a random-effects or mixed-effects model, metrics quantifying heterogeneity (e.g., using Q or I^2 statistics) are not always reported. Most reviews reported very low to moderate compliance on quantifying heterogeneity.

A common and prominent issues in exploring heterogeneity is that multiple explanatory variables are evaluated individually in separate models. This is an invalid approach because these explanatory variables may not be independent or because failure to simultaneously account for a factor may give rise to spurious results (e.g., via Simpson's paradox). A multifactorial analysis of moderators, which would address this issue, was reported only in a few meta-analyses. Gates [8] also mentioned that most meta-analyses did not correct for multiple testing when conducting subgroup analysis. This could reflect limitations imposed by software. For example, MetaWin, a commonly used software, does not allow multifactorial analyses. Additionally, Nakagawa & Santos [21] noted that meta-analytic data are often sparse and including all moderators in the model may result in great loss of sample size, making such analyses problematic [see 62]. This can be one of the reasons why multivariate analyses are less common [21]. However, given the increase in data and meta-data availability, and the availability of software with detailed documentation to run a multifactorial analysis of moderators [e.g., the R package metafor, 63], multifactorial analysis should become more feasible.

Sensitivity analysis. Sensitivity analyses are additional analyses conducted to test the robustness of a meta-analysis to methodological choices. They could involve explorations on how results change when removing influential points, or when including different types of information or weighting schemes, or when calculating different types of effect sizes. Sensitivity analysis can also be used to address issues related to non-independence, or at least to explore its consequences [64]. Across reviews, we observed that only a low percentage of meta-analyses reported having conducted a sensitivity analysis. This conclusion is likely conservative because we were not strict in our criteria and did not require the authors referring to these analyses explicitly as "sensitivity analysis". Although analysis for publication bias can be considered as a type of sensitivity analysis,

we followed previous reviews [e.g., 5] and quantified them as separate criteria. It is possible that some researchers may have run additional explorations that could be considered a sensitivity analysis in the earlier stages of a publication, but these were not stated explicitly in their final manuscripts or supplementary information. We encourage authors of meta-analyses to include the results of sensitivity analysis, to showcase the different types of limitations related to their dataset, and to quantify if different methodological choices affect their conclusions. This will enhance the robustness of their meta-analysis.

Publication bias. A general problem in scientific research is that significant results are more likely to be published. For meta-analysis, this may bias the meta-analyst towards discovering more significant effect sizes, which in turn may bias the conclusion of the meta-analysis. Meta-analysis has tools to identify the existence of publication bias (e.g., funnel plot) and to assess its impact (e.g., fail-safe number); these methods have pros and cons, and it is recommended to present results for at least two of them [11,21]. Despite the availability of specific methods, compliance to assess and report publication bias was low (<50%) in seven of the eight reviews that quantified this criterion.

A different type of publication bias in meta-analysis of ecology and evolution is temporal trends in effect sizes. For example, a decrease in the magnitude of effect sizes over time has been observed in various areas of ecology and evolution [65,66], although the existence of such a trend generally is debatable [67]. Possible non-biological causes for the decrease in the magnitude of effect sizes with time are time-lag bias, selective reporting, bias in the choice of research organisms with time, and changes in statistical methods with time [reviewed in 66]. Not accounting for temporal trends jeopardizes the stability of conclusions from meta-analyses [68]. Exploring

temporal trends in the magnitude of effect sizes could help address this issue and identify additional sources of heterogeneity in the effect sizes. A wide range of tools exist to analyze temporal trends in effect sizes. In the ecological meta-analyses reviewed by Pappalardo et al. [10], only 40% of meta-analyses reported the range of time analyzed in the main text, while 32% reported only the final year. From the publications reporting the full range, we observed an average time span of 41 years (min = 1, median = 34, max = 115). Cadotte et al. [6] also looked at the time span in the meta-analysis included in their review and found an average of 15 years and a maximum of 65 years. Despite the wide time span reported in many of the meta-analyses reviewed, the percent of meta-analysis that addressed temporal trends in effect size was very low (ranging from 1 to 5%). Koricheva et al. [66] describe a wide range of methods available to explore and to analyze temporal trends.

Methods for detecting and quantifying the effects of publication bias, such as regression- or correlation-based approach for analyzing the asymmetry of funnel plots, may encounter challenges in ecology and evolution due to heterogeneity and non-independence, two characteristics commonly associated with data in ecology and evolution [22]. To address this issue, Nakagawa et al. [22] proposed using what they referred to as "conditional residuals" from hierarchical models instead of observed effect sizes in analyzing funnel plots. This approach accounts for heterogeneity and non-independence by subtracting the fixed effects and random effects that model the heterogeneity and non-independence from the observed effect sizes. We recommend that readers consult Nakagawa et al. [22] for details of these methods.

Non-independence. Non-independence is common for biological data; if not addressed properly, non-independence can produce spurious results [69]. In particular, not properly

accounting for non-independence often leads to wrong estimates of standard error and thus invalid statistical inference. Non-independence may occur at the sampling level, i.e., non-independent within-study error or at the study level, i.e. non-independent random effect. The former may occur through using shared control or repeated measurements of the same individuals over time while the latter may occur because of phylogeny or similar environment shared by a set of studies. Our review of the meta-analyses compiled by Pappalardo et al. [10] showed a higher percent of papers (66%) that acknowledged some source of non-independence, compared with Archmiller et al. [15] and O'Dea et al. [12] that report between 44% and 14% respectively. When looking at the sources of the non-independence, the majority of the non-independence acknowledged was at the sampling level. For example, authors often recognize non-independence caused by using shared control or repeated measurements when calculating effect sizes. The most common approach to addressing sampling level non-independence was choosing a subset of the data (36%) or using the average of the non-independent measurements (50%). We did not observe any meta-analyses that tried to explicitly model the covariance of the sampling level non-independence even though formula for covariance have been derived for many scenarios of non-independence [70,71]. In contrast, non-independence arising from study-level correlation are much less recognized and addressed in meta-analyses. Only 14% of the meta-analyses from Pappalardo et al. [10] attempted to address study-level non-independence. This is mostly in the form of using a hierarchical model. However, study-level non-independence may arise in multiple ways in ecological meta-analyses [64]. In fact, we found that the number of effects far exceeds the number of papers in the meta-analyses we examined. Given that studies from the same source paper are more likely to share similar environments or methodology, it is very likely that study-level non-independence is common.

Thus, the relatively low proportion of published meta-analyses addressing such a source of non-independence is a source of concern.

A particularly common case of study-level non-independence arises from phylogeny. Closely related species may have similar traits that could be associated with similar responses; thus, data from different species may not be independent. In paired analysis of the same dataset using traditional and phylogenetic meta-analysis it has been shown that the magnitude of the effect size, the uncertainty around it (e.g., width of confidence interval), and its significance can change [17]. For example, Chamberlain et al. [17] report 40% of meta-analysis using random effects showing a difference in the significance of the effect size, with the majority of effects changing from significant to non-significant when a phylogenetic meta-analysis is used. The influence of phylogenetic relatedness on the outcome of meta-analysis has also been studied using simulations. Cinar et al., [72] found that under moderately strong phylogenetic relatedness, failing to account for species-level variance will generate biased estimates of overall means and poor coverage. This is troubling given that all the review papers showed very low compliance to controlling for phylogenetic non-independence.

How can we implement best practices?

As has been highlighted in many in multiple sessions at the 2020 Ecological Society of America meeting, there is a need for data integration at multiple scales, data synthesis, and training of young investigators on computer programming and the use of appropriate statistical tools. To address this gap, in the long term, we think that it is important to train ecologists on meta-analysis techniques. This could involve including meta-analysis topics in the curriculum of Ecology/Evolution graduate programs, which could be done as part of courses addressing statically methods and data analysis

or be required for prospectus exams. Alternatively, training could be given as short workshops. The data available for ecologists and the scope of the questions we need to answer are going to keep increasing, and we need to give all researchers the necessary tools for synthesis research.

For researchers to learn on their own, there are multiple resources available. Marc Lajeuneese has a YouTube channel (https://www.youtube.com/c/lajeunesselab) with multiple videos explaining techniques for the different steps to conduct a meta-analysis, and has also developed the R package *metagear* [13] that has functions to help with paper screening and data extraction (http://lajeunesse.myweb.usf.edu/metagear/metagear_basic_vignette.html). The Environmental Computing website (http://environmentalcomputing.net/meta-analysis/) provides tutorials to conduct meta-analysis with the R package *metafor* [63], and also general information on how to organize data that will be useful for meta-analysts (http://environmentalcomputing.net/data-entry/). The *metafor* website by Wolfgang Viechtbauer has detailed documentation and examples of data analysis and models to conduct a meta-analysis using *metafor* (https://www.metafor-project.org/doku.php/metafor). The CRAN task view for meta-analysis [73; https://cran.r-project.org/web/views/MetaAnalysis.html] provides a full list of R packages that have useful tools related with meta-analysis. Also available for R users is the online book *Doing meta-analysis with R: a hands-on guide* that is aimed at non-experts [74; https://bookdown.org/MathiasHarrer/Doing_Meta_Analysis_in_R/]. For Python users, there are also specific tools and resources focusing on meta-analysis [75,76]. For those who would prefer a friendly user interface, the software OpenMEE [77] provides advanced tools for meta-analysis in ecology and evolution (http://www.cebm.brown.edu/openmee/help.html). The Inter-Disciplinary Ecology and Evolution Lab (http://www.i-deel.org/links.html) provides several resources related

to meta-analysis and systematic reviews. Finally, Osenberg et al., (pers. comm.) are developing a series of meta-analysis lectures that will be freely available soon.

Our compilation highlights that the recommended Execution steps for a biological meta-analysis are the ones that need more attention. Now that specific guidelines are available with a focus on meta-analysis in Ecology and Evolution, authors can follow the PRISMA EcoEvo checklist [12] as a guide to plan their meta-analysis, and reviewers and editors to assess the quality of reporting in a meta-analysis. More importantly, improving the reporting quality and following guidelines will also improve the quality of the research. Although we showed a better overall performance in following Reporting criteria, the compliance was highly variable across reviews which suggest ample room for improvement. Based on the results of Pappalardo et al. [10], we also think that the type of confidence intervals used to measure the uncertainty around a mean effect size should be added to this checklist. Perhaps making the PRISMA EcoEvo checklist a mandatory reading step during paper submission for papers self-reported as meta-analysis, could help to 1) making explicit if a paper is not a statistical meta-analysis, 2) help to encourage good reporting, reproducibility, and overall quality. A key component to future meta-analyses and synthesis studies are data sharing and good data management practices [78].

The learning curve to conduct a meta-analysis and follow all the detailed steps may be steep and discouraging. The training opportunities mentioned above could help reduce the learning curve to implement recommended steps. Some researchers argue that we should not let the perfect be the enemy of the good [79]. We think that clear reporting of the steps to conduct the analysis, and data availability, at least can ensure follow up analysis. Including more of the recommended data-analysis steps will make the inferences more robust, the ultimate goal of a statistical tool.

Acknowledgments

We are very thankful to review authors Marc Cadotte, Scott Chamberlain, Steve Hovick, Julia Koricheva, Jessica Gurevitch, and João Paulo Romanelli for providing detailed additional methods or metadata associated with their publications. We are grateful to review author Shinichi Nakagawa for his prompt response to our request for additional information. We want to thank O'Dea et al., (2021) for sharing a draft of the PRISMA EcoEvo guidelines before it was published in its final form. PP is grateful for the encouragement made by an anonymous reviewer of the Pappalardo et al., (2020) publication to expand the analysis on the quality of reporting in meta-analysis in a separate publication. PP highly appreciates the feedback from Alyssa Gehman, Linsey Haram, Carrie Keogh, and Rachel Smith on an early draft of the manuscript.

References

1. Osenberg CW, St. Mary CM, Schmitt RJ, Holbrook SJ, Chesson P, Byrne B. Rethinking ecological inference: density dependence in reef fishes: Inference and density dependence. Ecol Lett. 2002 Nov 7;5(6):715–21.
2. Blankinship JC, Niklaus PA, Hungate BA. A meta-analysis of responses of soil biota to global change. Oecologia. 2011 Mar;165(3):553–65.
3. Thébault A, Mariotte P, Lortie CJ, MacDougall AS. Land management trumps the effects of climate change and elevated CO_2 on grassland functioning. Gibson D, editor. J Ecol. 2014 Jul;102(4):896–904.
4. Stewart G. Meta-analysis in applied ecology. Biol Lett. 2010 Feb 23;6(1):78–81.
5. Koricheva J, Gurevitch J. Uses and misuses of meta-analysis in plant ecology. Gómez-Aparicio L, editor. J Ecol. 2014 Jul;102(4):828–44.
6. Cadotte MW, Mehrkens LR, Menge DNL. Gauging the impact of meta-analysis on ecology. Evol Ecol. 2012 Sep;26(5):1153–67.
7. Lortie CJ. Formalized synthesis opportunities for ecology: systematic reviews and meta-analyses. Oikos. 2014 Aug;123(8):897–902.
8. Gates S. Review of methodology of quantitative reviews using meta-analysis in ecology. J Anim Ecol. 2002 Jul;71(4):547–57.
9. Gurevitch J, Curtis PS, Jones MH. Meta analysis in ecology. Adv Ecol Res. 2001;32:199–247.
10. Pappalardo P, Ogle K, Hamman EA, Bence JR, Hungate BA, Osenberg CW. Comparing traditional and Bayesian approaches to ecological meta- analysis. O'Hara RB, editor. Methods Ecol Evol. 2020 Jul 26;2041-210X.13445.

11. Nakagawa S, Noble DWA, Senior AM, Lagisz M. Meta-evaluation of meta-analysis: ten appraisal questions for biologists. BMC Biol. 2017 Dec;15(1):18.
12. O'Dea RE, Lagisz M, Jennions MD, Koricheva J, Noble DWA, Parker TH, et al. Preferred reporting items for systematic reviews and meta- analyses in ecology and evolutionary biology: a PRISMA extension. Biol Rev. 2021 Oct;96(5):1695–722.
13. Lajeunesse MJ. Facilitating systematic reviews, data extraction, and meta-analysis with the metagear package for R. Methods Ecol Evol. 2016;7:323–30.
14. Jack O. Wasey. PRISMAstatement: Plot flow charts according to the "PRISMA" statement [Internet]. 2019. Available from: https://CRAN.R-project.org/package=PRISMAstatement
15. ArchMiller AA, Bauer EF, Koch RE, Wijayawardena BK, Anil A, Kottwitz JJ, et al. Formalizing the definition of meta-analysis in *Molecular Ecology*. Mol Ecol. 2015 Aug;24(16):4042–51.
16. Beillouin D, Cardinael R, Berre D, Boyer A, Corbeels M, Fallot A, et al. A global overview of studies about land management, land- use change, and climate change effects on soil organic carbon. Glob Change Biol. 2022 Feb;28(4):1690–702.
17. Chamberlain SA, Hovick SM, Dibble CJ, Rasmussen NL, Van Allen BG, Maitner BS, et al. Does phylogeny matter? Assessing the impact of phylogenetic information in ecological meta-analysis: Phylogenetic meta-analysis. Ecol Lett. 2012 Jun;15(6):627–36.
18. Chaudhary VB, Walters LL, Bever JD, Hoeksema JD, Wilson GWT. Advancing Synthetic Ecology: A Database System to Facilitate Complex Ecological Meta-Analyses. Bull Ecol Soc Am. 2010 Apr;91(2):235–43.
19. Jennions MD, Kahn AT, Kelly CD, Kokko H. Meta-analysis and sexual selection: past studies and future possibilities. Evol Ecol. 2012 Sep;26(5):1119–51.
20. Lodi S, Godoy BS, Ortega JCG, Bini LM. Quality of meta- analyses in freshwater ecology: A systematic review. Freshw Biol. 2021 May;66(5):803–14.
21. Nakagawa S, Santos ESA. Methodological issues and advances in biological meta-analysis. Evol Ecol. 2012 Sep;26(5):1253–74.
22. Nakagawa S, Lagisz M, Jennions MD, Koricheva J, Noble DWA, Parker TH, et al. Methods for testing publication bias in ecological and evolutionary meta- analyses. Methods Ecol Evol. 2022 Jan;13(1):4–21.
23. Philibert A, Loyce C, Makowski D. Assessment of the quality of meta-analysis in agronomy. Agric Ecosyst Environ. 2012 Feb;148:72–82.
24. Roberts PD, Stewart GB, Pullin AS. Are review articles a reliable source of evidence to support conservation and environmental management? A comparison with medicine. Biol Conserv. 2006 Oct;132(4):409–23.
25. Romanelli JP, Meli P, Naves RP, Alves MC, Rodrigues RR. Reliability of evidence- review methods in restoration ecology. Conserv Biol. 2021 Feb;35(1):142–54.
26. Romanelli JP, Silva LGM, Gonçalves MCP, Naves RP, Almeida DRA, Resende AF, et al. Repeatability of the searching process in reviews of restoration outcomes. Restor Ecol [Internet]. 2021 Nov [cited 2022 Sep 18];29(8). Available from: https://onlinelibrary.wiley.com/doi/10.1111/rec.13496
27. Senior AM, Grueber CE, Kamiya T, Lagisz M, O'Dwyer K, Santos ESA, et al. Heterogeneity in ecological and evolutionary meta-analyses: its magnitude and implications. Ecology. 2016 Dec;97(12):3293–9.
28. Vetter D, Rücker G, Storch I. Meta- analysis: A need for well- defined usage in ecology and conservation biology. Ecosphere. 2013 Jun;4(6):1–24.
29. Shantz AA, Burkepile DE. Context-dependent effects of nutrient loading on the coral–algal mutualism. Ecology. 2014 Jul;95(7):1995–2005.
30. Yoon S, Read Q. Consequences of exotic host use: impacts on Lepidoptera and a test of the ecological trap hypothesis. Oecologia. 2016 Aug;181(4):985–96.
31. Gamfeldt L, Lefcheck JS, Byrnes JEK, Cardinale BJ, Duffy JE, Griffin JN. Marine

biodiversity and ecosystem functioning: what's known and what's next? Oikos. 2015 Mar;124(3):252–65.
32. Kuebbing SE, Nuñez MA. Negative, neutral, and positive interactions among nonnative plants: patterns, processes, and management implications. Glob Change Biol. 2015 Feb;21(2):926–34.
33. McDevitt-Irwin J, Iacarella J, Baum J. Reassessing the nursery role of seagrass habitats from temperate to tropical regions: a meta-analysis. Mar Ecol Prog Ser. 2016 Sep 28;557:133–43.
34. Wooliver R, Pfennigwerth AA, Bailey JK, Schweitzer JA. Plant functional constraints guide macroevolutionary trade- offs in competitive and conservative growth responses to nitrogen. Fox C, editor. Funct Ecol. 2016 Jul;30(7):1099–108.
35. Martinson HM, Fagan WF. Trophic disruption: a meta-analysis of how habitat fragmentation affects resource consumption in terrestrial arthropod systems. Hill J, editor. Ecol Lett. 2014 Sep;17(9):1178–89.
36. Feng Z, Rütting T, Pleijel H, Wallin G, Reich PB, Kammann CI, et al. Constraints to nitrogen acquisition of terrestrial plants under elevated CO_2. Glob Change Biol. 2015 Aug;21(8):3152–68.
37. He Q, Silliman BR. Consumer control as a common driver of coastal vegetation worldwide. Ecol Monogr. 2016 Aug;86(3):278–94.
38. Anderson JT. Plant fitness in a rapidly changing world. New Phytol. 2016 Apr;210(1):81–7.
39. R Core Team. R: A language and environment for statistical computing [Internet]. Vienna, Austria: R Foundation for Statistical Computing; 2020. Available from: https://www.R-project.org/
40. Wickham H, Seidel D. scales: Scale functions for visualization [Internet]. 2020. Available from: https://CRAN.R-project.org/package=scales
41. Gohel D. flextable: Functions for Tabular Reporting. R package version 0.6.10. [Internet]. 2021. Available from: https://CRAN.R-project.org/package=flextable
42. Daroczi G, Tsegelskyi R. pander: An R "Pandoc" Writer. R package version 0.6.5. [Internet]. 2022. Available from: https://CRAN.R-project.org/package=pander
43. Zhu H. kableExtra: Construct complex table with kable and pipe syntax [Internet]. 2021. Available from: https://CRAN.R-project.org/package=kableExtra
44. Wickham H, Bryan J. readxl: Read excel files [Internet]. 2019. Available from: https://CRAN.R-project.org/package=readxl
45. Neitmann T. ggcharts: Shorten the Distance from Data Visualization Idea to Actual Plot. R package version 0.2.1. [Internet]. 2020. Available from: https://CRAN.R-project.org/package=ggcharts
46. Wickham H. Welcome to the tidyverse. J Open Source Softw. 2019;4(43):1686.
47. Albert A, Auffret AG, Cosyns E, Cousins SAO, D'hondt B, Eichberg C, et al. Seed dispersal by ungulates as an ecological filter: a trait-based meta-analysis. Oikos. 2015 Sep;124(9):1109–20.
48. Yuan ZY, Chen HYH. Decoupling of nitrogen and phosphorus in terrestrial plants associated with global changes. Nat Clim Change. 2015 May;5(5):465–9.
49. Englund G, Sarnelle O, Cooper SD. The importance of data-selection criteria: meta-analysis of stream predation experiments. Ecology. 1999 Jun;80(4):1132–41.
50. Culina A, van den Berg I, Evans S, Sánchez-Tójar A. Low availability of code in ecology: A call for urgent action. PLOS Biol. 2020 Jul 28;18(7):e3000763.
51. Carpenter SR, Armbrust EV, Arzberger PW, Chapin FS, Elser JJ, Hackett EJ, et al. Accelerate Synthesis in Ecology and Environmental Sciences. BioScience. 2009 Sep;59(8):699–701.
52. Powers SM, Hampton SE. Open science, reproducibility, and transparency in ecology. Ecol Appl [Internet]. 2019 Jan [cited 2022 Jul 17];29(1). Available from:

https://onlinelibrary.wiley.com/doi/10.1002/eap.1822
53. Moher D, Liberati A, Tetzlaff J, Altman DG, Group and the P. Preferred Reporting Items for Systematic Reviews and Meta-Analyses: The PRISMA Statement. Ann Intern Med. 2009;151(4):264–9.
54. Chalmers I, Haynes B. Reporting, updating, and correcting systematic reviews ofthe effects ofhealth care. 1994;309:4.
55. Tunis AS, McInnes MDF, Hanna R, Esmail K. Association of Study Quality with Completeness of Reporting: Have Completeness of Reporting and Quality of Systematic Reviews and Meta-Analyses in Major Radiology Journals Changed Since Publication of the PRISMA Statement? Radiology. 2013 Nov;269(2):413–26.
56. Maticic K, Krnic Martinic M, Puljak L. Assessment of reporting quality of abstracts of systematic reviews with meta-analysis using PRISMA-A and discordance in assessments between raters without prior experience. BMC Med Res Methodol. 2019 Dec;19(1):32.
57. Panic N, Leoncini E, de Belvis G, Ricciardi W, Boccia S. Evaluation of the Endorsement of the Preferred Reporting Items for Systematic Reviews and Meta-Analysis (PRISMA) Statement on the Quality of Published Systematic Review and Meta-Analyses. Derrick GE, editor. PLoS ONE. 2013 Dec 26;8(12):e83138.
58. Nawijn F, Ham WHW, Houwert RM, Groenwold RHH, Hietbrink F, Smeeing DPJ. Quality of reporting of systematic reviews and meta-analyses in emergency medicine based on the PRISMA statement. BMC Emerg Med. 2019 Dec;19(1):19.
59. Kambach S, Bruelheide H, Gerstner K, Gurevitch J, Beckmann M, Seppelt R. Consequences of multiple imputation of missing standard deviations and sample sizes in meta- analysis. Ecol Evol. 2020 Oct;10(20):11699–712.
60. Shantz AA, Lemoine NP, Burkepile DE. Nutrient loading alters the performance of key nutrient exchange mutualisms. Knops J, editor. Ecol Lett. 2016 Jan;19(1):20–8.
61. Gurevitch J, Hedges LV. Statistical issues in ecological meta-analyses. Ecology. 1999;80(4):1142–9.
62. Trager MD, Bhotika S, Hostetler JA, Andrade GV, Rodriguez-Cabal MA, McKeon CS, et al. Benefits for Plants in Ant-Plant Protective Mutualisms: A Meta-Analysis. Raine NE, editor. PLoS ONE. 2010 Dec 22;5(12):e14308.
63. Viechtbauer W. Conducting Meta-Analyses in R with the metafor Package. J Stat Softw [Internet]. 2010 [cited 2019 Jan 10];36(3). Available from: http://www.jstatsoft.org/v36/i03/
64. Noble DWA, Lagisz M, O'dea RE, Nakagawa S. Nonindependence and sensitivity analyses in ecological and evolutionary meta-analyses. Mol Ecol. 2017 May;26(9):2410–25.
65. Jennions MD, Møller AP. Relationships fade with time: a meta-analysis of temporal trends in publication in ecology and evolution. Proc R Soc Lond B Biol Sci. 2002 Jan 7;269(1486):43–8.
66. Koricheva J, Jennions MD, Lau J. Temporal trends in effect sizes: causes, detection, and implications. In: Handbook of meta-analysis in ecology and evolution. Princeton, New Jersey: Princeton University Press; 2013. (Chapter 5).
67. Costello L, Fox JW. Decline effects are rare in ecology. Ecology. 2022;103(6):e3680.
68. Koricheva J, Kulinskaya E. Temporal instability of evidence base: a threat to policy making? Trends Ecol Evol. 2019 Oct;34(10):895–902.
69. Song C, Peacor SD, Osenberg CW, Bence JR. An assessment of statistical methods for nonindependent data in ecological meta-analyses. Ecology. 2020;101(12):e03184.
70. Gleser LJ, Olkin I. Stochastically dependent effect sizes. In: Handbook of research synthesis and meta-analysis. New York: The Russell Sage Foundation; 1994. (2nd edition).
71. Lajeunesse MJ. On the meta-analysis of response ratios for studies with correlated and multi-group designs. Ecology. 2011 Nov;92(11):2049–55.

72. Cinar O, Nakagawa S, Viechtbauer W. Phylogenetic multilevel meta- analysis: A simulation study on the importance of modelling the phylogeny. Methods Ecol Evol. 2022 Feb;13(2):383–95.
73. Dewey M, Viechtbauer W. CRAN Task View: Meta-Analysis [Internet]. 2022. Available from: https://CRAN.R-project.org/view=MetaAnalysis
74. Harrer M. Doing meta-analysis with R: a hands-on guide. First edition. Boca Raton: CRC Press; 2022. 1 p.
75. Masoumi S, Shahraz S. Meta-analysis using Python: a hands-on tutorial. BMC Med Res Methodol. 2022 Dec;22(1):193.
76. Paracha UZ. Basics of meta-analysis with basic steps in Python (Programming language). Independently published; 2021. 116 p.
77. Wallace BC, Lajeunesse MJ, Dietz G, Dahabreh IJ, Trikalinos TA, Schmid CH, et al. OpenMEE: Intuitive, open- source software for meta- analysis in ecology and evolutionary biology. Poisot T, editor. Methods Ecol Evol. 2017 Aug;8(8):941–7.
78. Hampton SE, Strasser CA, Tewksbury JJ, Gram WK, Budden AE, Batcheller AL, et al. Big data and the future of ecology. Front Ecol Environ. 2013 Apr;11(3):156–62.
79. Munafò M. Don't let the perfect be the enemy of the good. PLOS Biol. 2021 Jul 15;19(7):e3001327.

Supporting Information captions

~~**Appendix A**. Details on the information extracted from each paper for each performance criterion. For data from Pappalardo et al. [10], we indicated when the data was collected in this study by re-reviewing their compilation of ecological meta analyses. When the number of publications complying (or not complying) with one of the criteria was reported, we used that information to calculate the percentage of papers complying; in other cases the reviews directly reported the information as a percentage. In a few papers in which we had the original review data for each criterion (e.g., [15]), we summed the number of papers complying with each criterion, and then calculated the percentage of compliance based on the total number of papers relevant for that criterion.~~

Appendix A. Details on the information extracted from each paper for each performance criterion.

Details on the information extracted from each paper for each performance criterion. For data from Pappalardo et al. (2020), we indicated when the data was collected in this study by re-reviewing their compilation of ecological meta-analyses with the tag “added”. When the number of publications complying (or not complying) with one of the criteria was reported, we used that information to calculate the percentage of papers complying; in other cases the reviews directly reported the information as a percentage. In a few papers in which we had the original review data for each criterion (e.g., Archmiller et al., 2015), we summed the number of papers complying with each criterion, and then calculated the percentage of compliance based on the total number of papers relevant for that criterion.

Fig S1. PRISMA diagram.

The PRISMA diagram details the relevant literature sources identified, screened, excluded, assessed, and selected for the final analysis.

Fig S2. Distribution of paper overlap for Reporting criteria.

Distribution of the number of papers shared between reviews for all the Reporting criteria combined.

Fig S3. Distribution of paper overlap for Execution criteria.

Distribution of the number of papers shared between reviews for all the Execution criteria combined.

Table S1. Number of meta-analyses per journal that has been included in the meta-anlyses reviews. Because the distribution is strongly right skewed (with most journals publishing a few meta-analyses), we display only the journals with at least 5 meta-analyses.

Fig S4. Percent of papers complying with Reporting criteria by review discipline.

The percent of papers complying with each criterion is plotted for each synthesis paper. The colors indicate different subdisciplines of the review papers. The Review ID corresponds to the papers listed in Table 1 of the main manuscript.

Fig S5. Percent of papers complying with Execution criteria by review discipline.

The percent of papers complying with each criterion is plotted for each synthesis paper. The colors indicate different subdisciplines of the review papers. The Review ID corresponds to the papers listed in Table 1 of the main manuscript.

Fig S6. Percent of papers complying with Reporting criteria as a function of the time period analyzed by the review paper.

Each panel represents a criterion. The line segment indicates the time period covered by each of the review papers that addressed a particular criterion.

Fig S7. Percent of papers complying with Execution criteria as a function of the time period analyzed by the review paper.

Each panel represents a criterion. The line segment indicates the time period covered by each of the review papers that addressed a particular criterion.

Data availability statement

All the code and data files used for the analysis are provided in the Supplementary material.

Author’s contributions

Following CREDIT guidelines: Conceptualization, P.P., C.W.O., B.H., & C.S.; Methodology, P.P., C.W.O., & C.S.; Investigation, P.P. & CS; Writing – Original Draft, P.P.; Writing – Review & Editing, P.P., C.O., B.H., & C.S.; Funding Acquisition, C.W.O.

ORCID

Paula Pappalardo https://orcid.org/0000-0003-0853-7681

Chao Song: https://orcid.org/0000-0001-8225-4490

Bruce A. Hungate: https://orcid.org/0000-0002-7337-1887

Craig W. Osenberg: https://orcid.org/0000-0003-1918-7904

Figure 1 Click here to access/download;Figure;Fig_1.tif

R Notebook.pdf

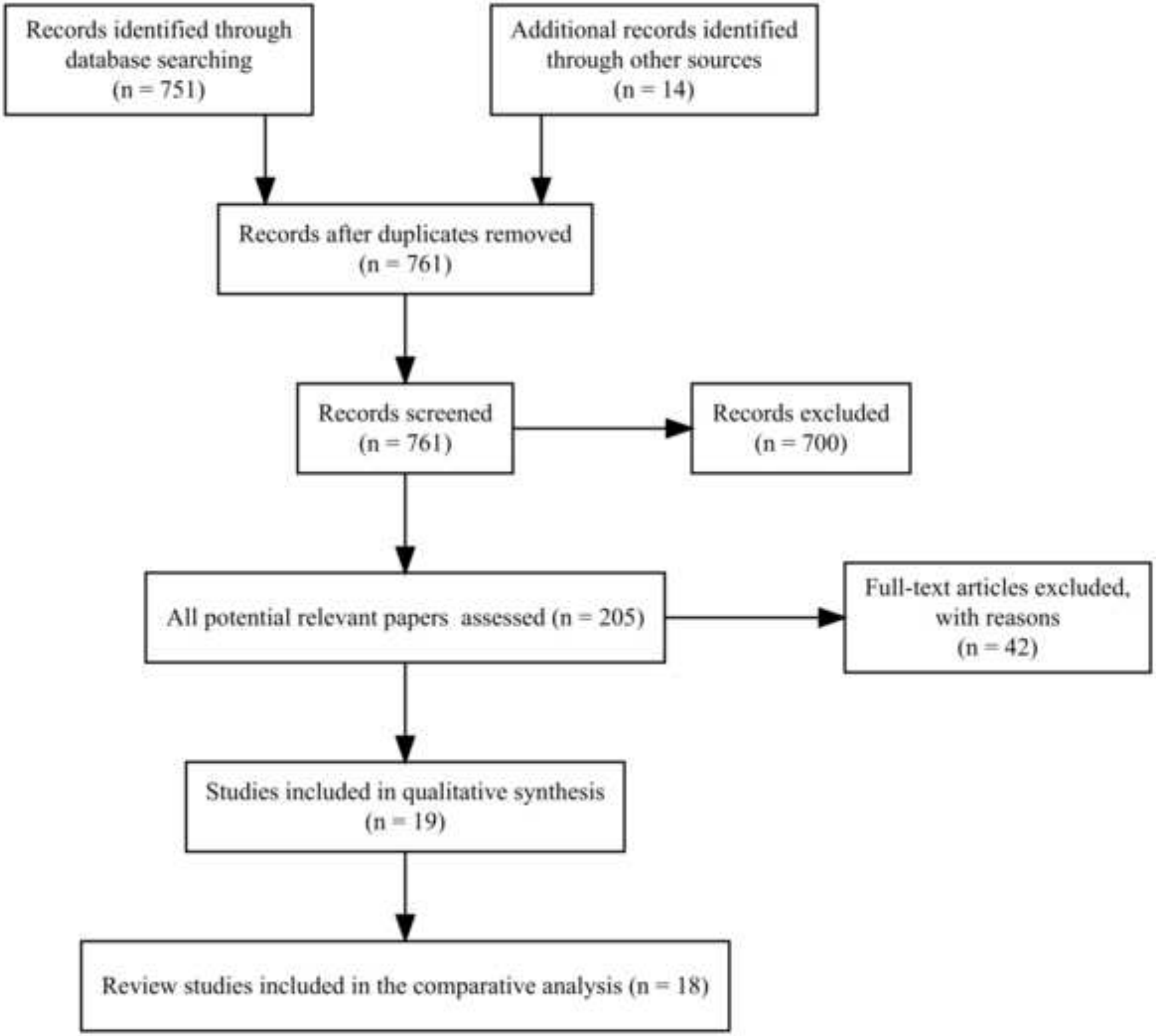

Figure 2

Click here to access/download;Figure;Fig_2.tif

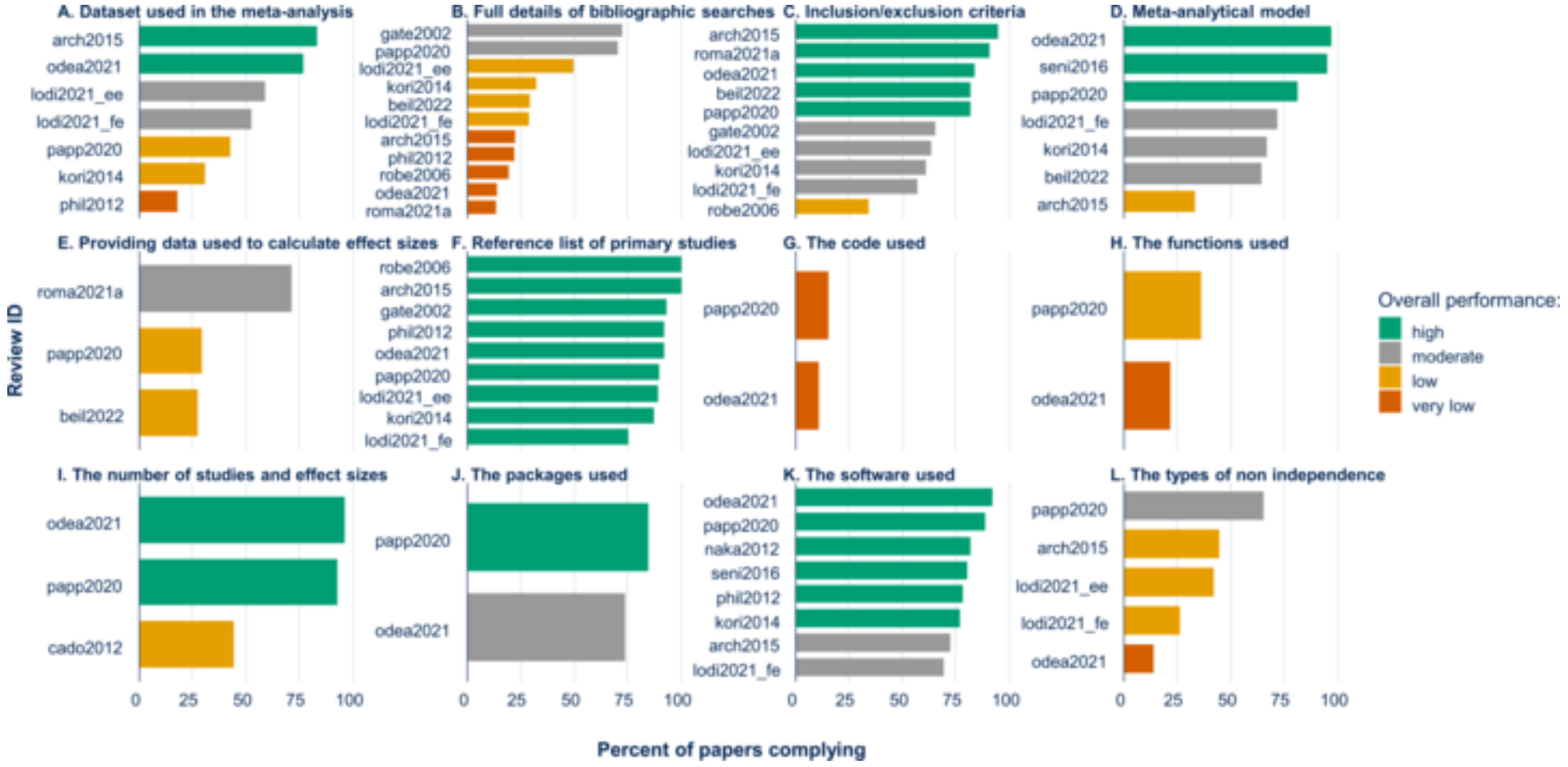

Fig_1.pdf

Figure 3

Click here to access/download;Figure;Fig_3.tif

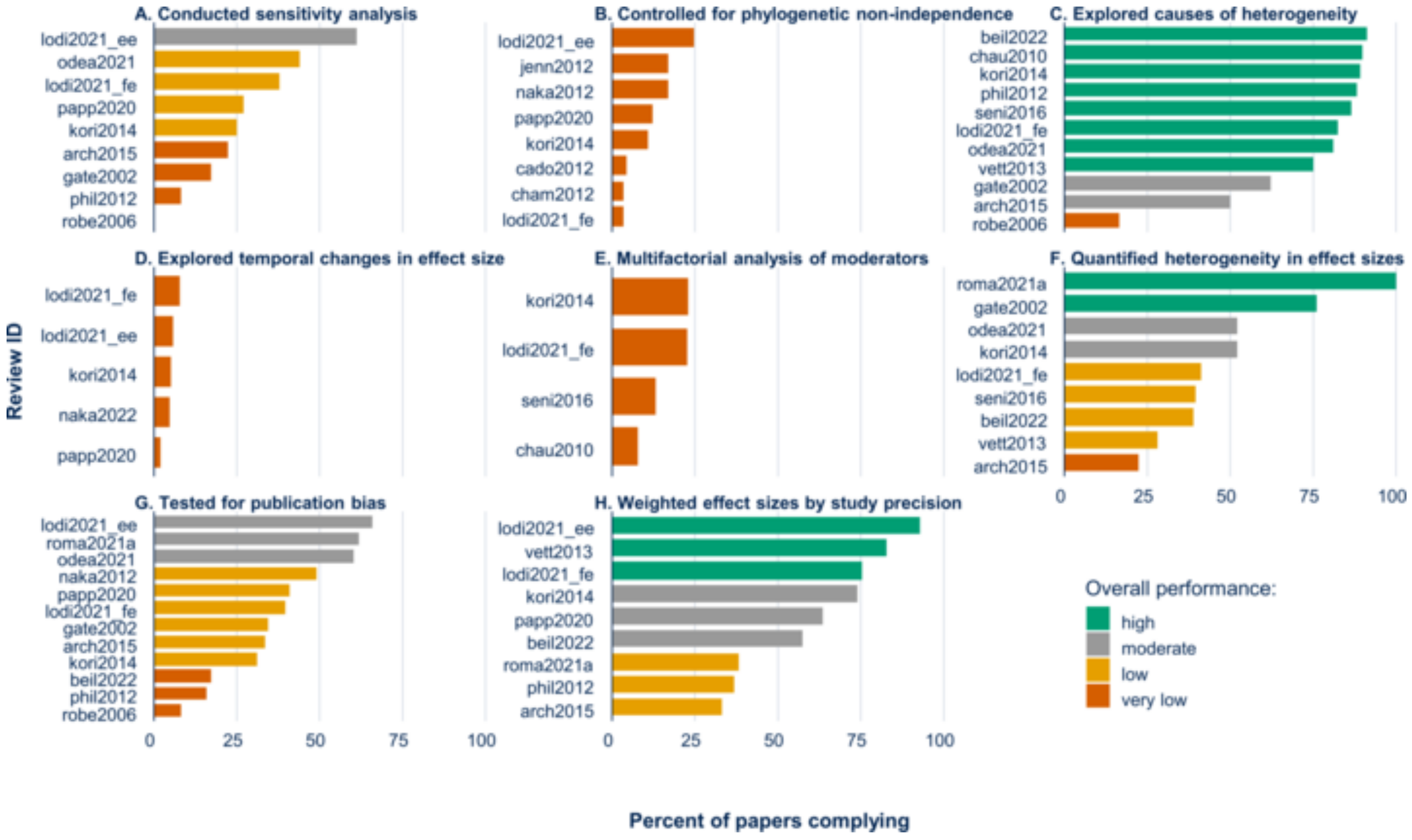

Fig_2.pdf

Figure 4

Click here to access/download;Figure;Fig_4.tif

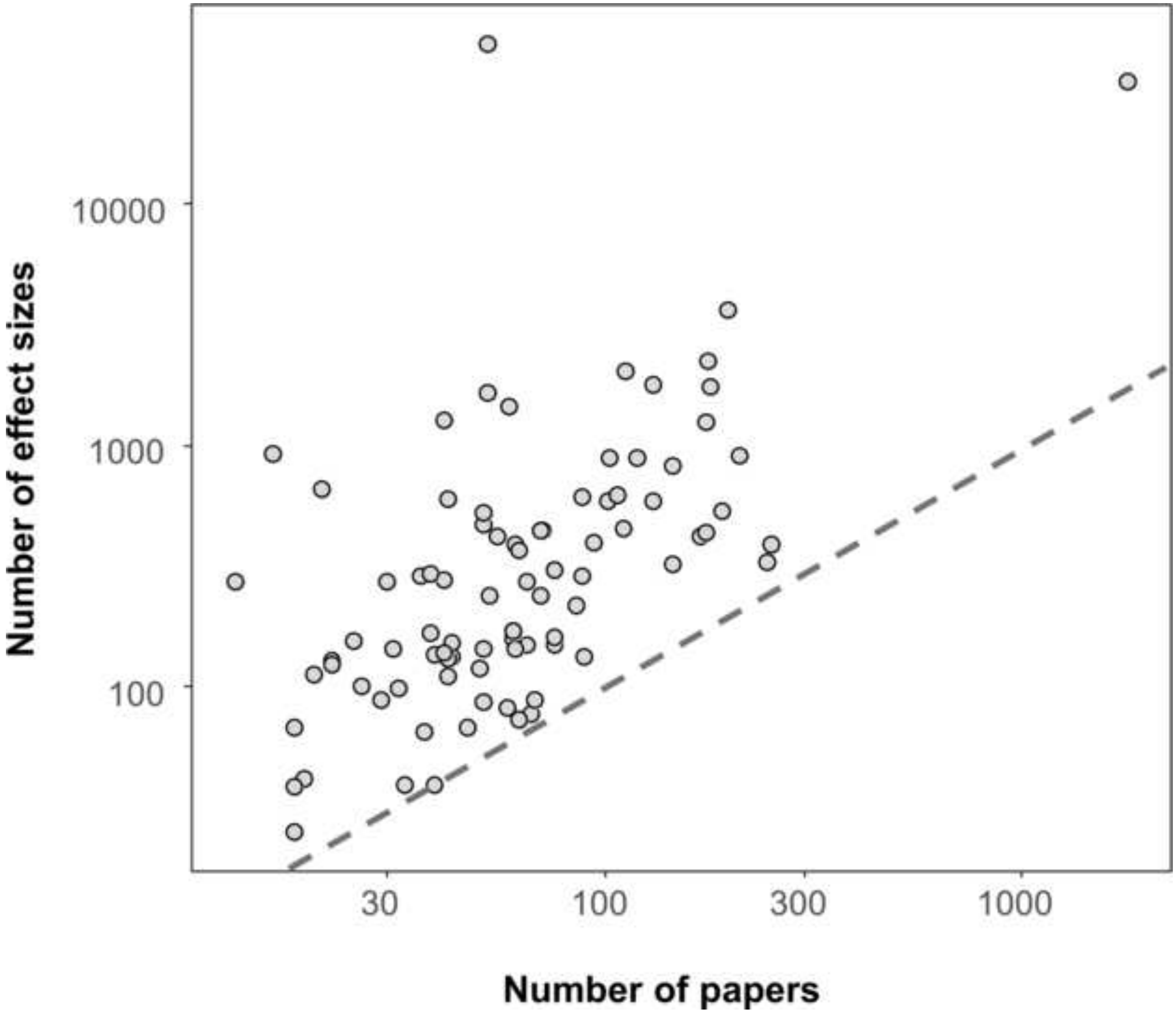

Fig_3.pdf

Figure 5

Click here to access/download;Figure;Fig_5.tif

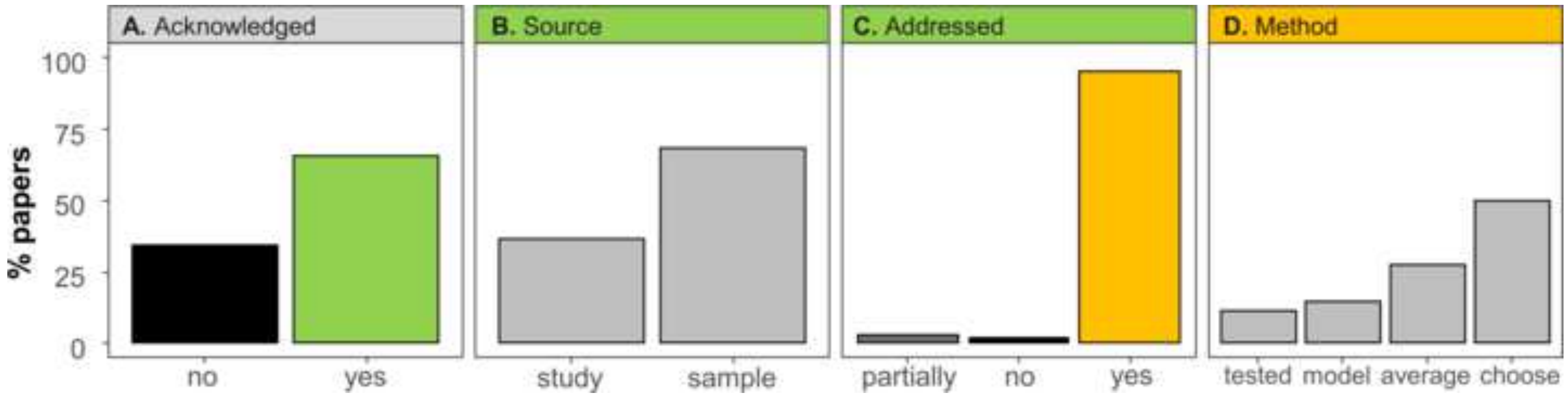

Fig4.pdf

Appendix A

Click here to access/download
Supporting Information
Appendix-criteria.docx.pdf

Click here to access/download
Supporting Information
QualityOfReporting_SupplementaryMaterial_2022-09.pdf

Click here to access/download
Supporting Information
QualityOfReporting_2022-09.pdf

Supporting Information - list of references in review papers

Click here to access/download
Supporting Information
DataFiles_list-of-references-in-Reviews.xlsx

Supporting Information - new data from Pappalardo et al meta analyses

Click here to access/download
Supporting Information
DataFiles_new-data.xlsx

Supporting Information - compilation of data from previous review papers

Click here to access/download
Supporting Information
DataFiles_compilation-of-previous-review-papers.xlsx

Click here to access/download
Supporting Information
DataFiles_journal-names-dictionary.csv

Click here to access/download
Supporting Information
PRISMA_2020_checklist.docx
