# Supplementary material for: A meta-evaluation of the quality of reporting and execution in ecological meta-analyses
Source: PLoS One. 2023 Oct 12;18(10):e0292606. doi: 10.1371/journal.pone.0292606 (PMC10569516; doi:10.1371/journal.pone.0292606)
Supplement: Attachment — Submitted filename: 2023-09-22_Response-to-reviewers.docx [file pone.0292606.s021.docx]

*[Note – all our responses are embedded here in blue italicized font; line numbers in the response refer to the final (clean) manuscript version, not to the track-changes version]*

**Journal Requirements:**

Please review your reference list to ensure that it is complete and correct. If you have cited papers that have been retracted, please include the rationale for doing so in the manuscript text, or remove these references and replace them with relevant current references. Any changes to the reference list should be mentioned in the rebuttal letter that accompanies your revised manuscript. If you need to cite a retracted article, indicate the article’s retracted status in the References list and also include a citation and full reference for the retraction notice.


*--- Response: We have reviewed the references, fixed the error on the text found by Reviewer #2, and made no changes to the overall list of references.*


**Response To Reviewers' comments**:

**Reviewer #2:**

Dear authors and Editor,
I think that the reviewed version of the manuscript “A meta-evaluation of the quality of reporting and execution in ecological meta-analyses” included and/or rebutted reasonably all the points observed by all reviewers. I read only small points (most are typos):
l. 107: "PDFs of".

*--- Response: Fixed. Thank you for catching this typo.*

l. 114: use ";" instead of ":".

*--- Response: Done.*

l. 129-130: "Romanelli et al. Reviews [14, 15]".

*--- Response: Fixed, thank you for catching this.*

l. 205: "[...] across species);".

*--- Response: Parenthesis added, thank you for catching this.*

l. 277-278: I understood the message here, but in my opinion, the example here should be "e.g., multiple effect sizes per publication". I will extend this minor point because there are not many corrections to indicate in this study... I (gladly) ended more thinking than suggesting corrections here again.
Several independent experiments can be published in a single paper. There are prospective meta-analyses where a set of independent experiments are conducted to be combined with meta-analytical tools (Ioannidis 2017. DOI: 10.1136/bjsports-2017-097621; e.g. Kawaguchi 2013. DOI: 10.1264/jsme2.me13014). This would not be an "explicit" violation of the independence of effect sizes (I am thinking here of effect size multiplicity only; Lopez-Lopez et al. 2018. DOI: 10.1002/jrsm.1310). Sure, we can think of the possibility of effect sizes being published by the same research group as being more similar than those from different research groups, but this would be a dependence above the study level.

*--- Response: This is a good point, we edited the text as suggested.*

l. 351: "PRISMA".

*--- Response: Fixed. Thank you for catching this typo.*

l. 361-362: Not needed. L. 360-361 could be joined to l. 363.
l. 549: "framework),".
*--- Response: Edited as suggested.*

Congratulations to the authors for conducting this study! It was a pleasure to read it. Looking forward to the published version to recommend it in classes and citing.

*--- Response: We really appreciate all your time to re-review the manuscript and all the suggestions to make it better.*

Best regards,
Jean C. G. Ortega

**Reviewer #3:**

Thank you for promptly considering the suggestions made to improve your manuscript. Find below some other small details, mostly associated with language.
*--- Response: Thank you very much for all the suggestions and your time to re-review and help us improve our manuscript.*

L23: Each of the 18 review papers

*--- Response: Edited as suggested.*

L29: … a wide variation

*--- Response: Edited as suggested.*

L63-65: Sentence is confusing. I suggest changing to:
“The quality of a meta-analysis also is affected by how well the study is implemented. We refer to this proper implementation as execution quality, which is the extent to which the analyses conform to expert recommendation.”

*--- Response: Edited as suggested.*

L111: change “(…) in restoration ecology; in [14] (...)” to “Romanelli et al. [14] describe (...)”

*--- Response: Edited as suggested.*

L116: Meta-analysis should be plural

*--- Response: Edited as suggested.*

L117: separate should be singular

*--- Response: Edited as suggested.*

L159: provided should be in the past tense

*--- Response: Edited as suggested.*

L217-218: change “is provided” to “are provided”

*--- Response: Edited as suggested.*

L377: change : to ,

*--- Response: Edited as suggested.*

L410: remove “as” in “publication bias can be considered as a type”

*--- Response: Edited as suggested.*

L354-455: use “analysis concerned the method…” or “analysis was associated with”

*--- Response: Edited as the second option.*
